# Multi-omics evaluation of the prognostic value and immune signature of FCN1 in pan-cancer and its relationship with proliferation and apoptosis in acute myeloid leukemia

**DOI:** 10.3389/fgene.2024.1425075

**Published:** 2024-07-29

**Authors:** Fangfang Zhong, Lijun Song, Hao li, Jing Liu, Chunyan Liu, Qulian Guo, Wenjun Liu

**Affiliations:** ^1^ Department of Pediatrics, Children Hematological Oncology and Birth Defects Laboratory, The Affiliated Hospital of Southwest Medical University, Sichuan Clinical Research Center for Birth Defects, Luzhou, Sichuan, China; ^2^ Department of Pediatrics, Hejiang County People’s Hospital, Luzhou, Sichuan, China

**Keywords:** FCN1, macrophages, AML, apoptosis, biomarkers

## Abstract

**Background:**

The FCN1 gene encodes the ficolin-1 protein, implicated in the pathogenesis of various diseases, though its precise role in tumorigenesis remains elusive. This study aims to elucidate the prognostic significance, immune signature, and treatment response associated with FCN1 across diverse cancer types.

**Methods:**

Employing multi-omics data, we conducted a comprehensive assessment, encompassing tissue-specific and single-cell-specific expression disparities, pan-cancer expression patterns, epigenetic modifications affecting FCN1 expression, and the immune microenvironment. Our investigation primarily focused on the clinical prognostic attributes, immune profiles, potential molecular mechanisms, and candidate therapeutic agents concerning FCN1 and acute myeloid leukemia (AML). Additionally, *in vitro* experiments were performed to scrutinize the impact of FCN1 knockdown on cell proliferation, apoptosis, and cell cycle dynamics within the AML cell line U937 and NB4.

**Results:**

FCN1 expression exhibits widespread dysregulation across various cancers. Through both univariate and multivariate Cox regression analyses, FCN1 has been identified as an independent prognostic indicator for AML. Immunological investigations elucidate FCN1’s involvement in modulating inflammatory responses within the tumor microenvironment and its correlation with treatment efficacy. Remarkably, the deletion of FCN1 influences the proliferation, apoptosis, and cell cycle dynamics of U937 cells and NB4 cells.

**Conclusion:**

These findings underscore FCN1 as a promising pan-cancer biomarker indicative of macrophage infiltration, intimately linked with the tumor microenvironment and treatment responsiveness, and pivotal for cellular mechanisms within AML cell lines.

## 1 Introduction

Cancer represents a formidable global health challenge, exerting a significant impact on populations worldwide (Wang et al., 2023). Recent projections by the American Cancer Society estimate approximately two million new cancer cases and 610,000 cancer-related deaths in the United States for 2024 (Siegel et al., 2024). Despite notable strides in treatment modalities, the burden of cancer persists globally (Sabado et al., 2017). The advent of immunotherapy marks a transformative milestone in cancer management, particularly with breakthroughs in immune checkpoint inhibitors and chimeric antigen receptor (CAR) T cell therapy (Feins et al., 2019; Wang et al., 2020; Sterner and Sterner, 2021). While clinical outcomes underscore remarkable efficacy across various malignancies such as non-small cell lung cancer, triple-negative breast cancer, ovarian cancer, and leukemia, challenges persist, including tumor heterogeneity, treatment-related adverse effects, and economic considerations (Bardia et al., 2021; Hu et al., 2021; Park et al., 2021; Zsiros et al., 2021). The emergence of pan-cancer analytical approaches enhances our understanding of tumor diversity, offering a macroscopic viewpoint to unveil underlying mechanisms of tumorigenesis. By elucidating shared immunological traits across cancers, these methodologies illuminate potential therapeutic targets and avenues for innovation in cancer treatment strategies.

The FCN1 gene encodes ficolin-1, a plasma protein classified within the immunoglobulin superfamily (Chen et al., 2023). It plays a pivotal role in the body’s immune defense, particularly in the identification and elimination of pathogens (Okuzaki et al., 2017). Through interaction with carbohydrate structures on pathogen surfaces, the FCN1 protein initiates immune responses, including complement system activation and inflammation promotion (Munthe-Fog et al., 2012). Given its significance in disease pathogenesis, FCN1 gene polymorphisms have been associated with autoimmune disorders such as systemic lupus erythematosus and rheumatoid arthritis, highlighting its involvement in autoimmunity. Additionally, the FCN1 protein plays a crucial role during infection and inflammation, facilitating pathogen clearance, including bacteria, fungi, and viruses, by recognizing and binding to their surface carbohydrate structures (Vander et al., 2007; Addobbati et al., 2016; MacDonald et al., 2021).

Considering the pivotal role of inflammation in the tumor microenvironment, understanding FCN1’s function in autoimmune diseases, infection, and inflammation is well-established, but its involvement in cancer remains unclear. Hence, our study aims to comprehensively analyze FCN1 in pan-cancer settings from a multi-omics perspective, focusing on its expression variances, genetic alterations, immunological attributes, and predictive potential for immunotherapy. Specifically, we concentrate on investigating FCN1’s clinical prognostic relevance, distinct immunological features, potential functional mechanisms, and drug targeting predictions in acute myeloid leukemia (AML). Furthermore, we assess its probable impact on AML cell proliferation, apoptosis, and cycle regulation through cellular experiments. By elucidating FCN1’s role in tumor immunology, our research aims to provide insights into its potential mechanisms and offer avenues for clinical diagnosis and immunotherapeutic exploration.

## 2 Materials and methods

### 2.1 Datasets acquisition

Integrating the normalized expression profiles of The Cancer Genome Atlas (TCGA) and the Genotype-Tissue Expression (GTEx) databases, using the GISTIC2.0 method to estimate pan-cancer copy number variation (CNV) of gene expression, DNA methylation profiles, Therapeutically Applicable Research To Generate Effective Treatments (TARGET) AML gene expression profiles and clinical data were downloaded from UCSC Xena browser (https://xenabrowser.net/) (Mermel et al., 2011). Human normal tissue expression profiles were sourced from the Human Protein Atlas (HPA) database (https://www.proteinatlas.org/), while cancer cell line expression profile data were obtained from the Cancer Cell Line Encyclopedia (CCLE). The Beat AML cohort was derived from prior research (Bottomly et al., 2022). Microsatellite instability (MSI), Homologous recombination deficiency (HRD), and Loss of heterozygosity (LOH) data were extracted from previous studies (Bonneville et al., 2017; Thorsson et al., 2018). Additionally, we acquired additional datasets from the Gene Expression Omnibus (GEO) database to assess FCN1 expression and its association with drug sensitivity. For detailed information on all datasets, please refer to Supplementary Tables S1-S3.

### 2.2 Expression landscape, genetic changes and prognostic value of FCN1 in pan-cancer

The expression profiles of TCGA and GTEx were integrated, and the differential expression of FCN1 mRNA and transcripts across 33 types of tumors and normal tissues was compared. Validation of FCN1’s expression in multiple tissues was conducted using the Gene Expression database of Normal and Tumor tissues (GENT2, http://gent2.appex.kr/gent2/) (Park et al., 2019). Furthermore, the University of Alabama at Birmingham Cancer Data Analysis Portal (UALCAN, https://ualcan.path.uab.edu/) was utilized to assess FCN1 protein expression levels in tumor and normal samples (Chandrashekar et al., 2022). The TISIDB database (http://cis.hku.hk/TISIDB/index.php) was used to evaluate the expression levels of FCN1 in different subtypes. cBioPortal (http://www.cbioportal.org/) facilitated the examination of FCN1 genomic mutations, amplifications, and deep deletions in pan-cancer datasets. Additionally, CNV levels and methylation differences of FCN1 across various cancers were evaluated. Prognostic relevance of FCN1 methylation was assessed through Kaplan-Meier (KM) curves using the Tumor Immune Dysfunction and Elimination (TIDE) Methylation module (Jiang et al., 2018). Pan-cancer receiver operating characteristic (ROC) curves were generated using the R “pROC” package (Robin et al., 2011). Patients were stratified based on optimal FCN1 expression cutoff values, and survival outcomes were compared using KM curves, with the R “survival” and “survminer” packages employed for their generation (Shi et al., 2023). Univariate Cox regression analysis, conducted using the R packages “survival” and “forestplot”, evaluated the association of FCN1 expression with pan-cancer overall survival (OS), disease-specific survival (DSS), progression-free interval (PFI), and disease-free interval (DFI) (Li et al., 2023).

### 2.3 Gene set enrichment analysis (GSEA) and correlation analysis

The GeneMANIA online database (http://genemania.org/) was utilized to predict the gene function of FCN1, while the STRING database was employed to construct a protein-protein interaction network for FCN1 (Franz et al., 2018; Szklarczyk et al., 2023). To elucidate FCN1-related pathways, tumor samples were categorized based on median FCN1 expression, followed by Gene Set Enrichment Analysis (GSEA) using the Hallmarks gene set (h.all.v7.5.1. symbols.gmt) from the Molecular Signatures Database (MSigDB, https://www.gsea-msigdb.org/gsea/msigdb). Additionally, leveraging the 14 functional states of malignant tumors from CancerSEA (http://biocc.hrbmu.edu.cn/CancerSEA/home.jsp), we applied the “z-score” algorithm across these gene sets using the R package “GSVA” (Hänzelmann et al., 2013). Pearson correlation analysis was then used to assess the statistical relationship between FCN1 expression and the z-scores of these gene sets. Furthermore, the top 30% of samples with the highest FCN1 expression were designated as the high expression group, while the bottom 30% were categorized as the low expression group. Differences in GSVA scores of 73 Kyoto Encyclopedia of Genes and Genomes (KEGG) database metabolic gene sets between the FCN1 high and low expression groups were compared using the limma package.

### 2.4 Pan-cancer analyses of the immunological roles of FCN1

We investigated the correlation between FCN1 and immune-related genes such as MHC, chemokine receptors, chemokines, immunosuppressive genes, and immunostimulatory genes across various cancer types. Additionally, correlations between FCN1 and previously identified immune checkpoint markers were examined at the mRNA level. Furthermore, the study evaluated the differential expression of FCN1 across six immunological subtypes (C1: Wound healing; C2: IFN-γ dominant; C3: Inflammatory; C4: Lymphocyte-depleted; C5: Immunologically quiet; C6: TGF-β dominant) using the TISIDB subtype module. The relative proportions of infiltrating immune cells were quantified using the xCell, EPIC, MCPCOUNTER, and CIBERSORT-ABS algorithms, with Spearman’s correlation coefficients utilized to explore the association between FCN1 expression and the relative abundance of different types of infiltrating immune cells. Moreover, the TIMER2.0 database (http://timer.cistrome.org/) was employed to investigate the correlation between FCN1 expression and macrophage infiltration. Single-cell resolution analysis of FCN1 in pan-cancer was conducted using Tumor Immune Single-cell Hub 2 (TISCH2) (Han et al., 2023). We generated a heatmap to depict the FCN1 expression profile in different cancer types at the single-cell level. Using the Uniform Manifold Approximation and Projection (UMAP) technique, we visualized these high-dimensional data in a two-dimensional heatmap format. For the spatial transcriptome data, we used deconvolution analysis to accurately assess the cellular composition of each spot on the 10xVisium slide and to establish a comprehensive scRNA reference library. The enrichment score matrix was generated using the get_enrichment_matrix and enrichment_analysis functions in the Cottrazm package, and the SpatialFeaturePlot function in the Seurat package was used to visualize the enrichment score of each cell type (Mangiola et al., 2021). In addition, Spearman correlation analysis was performed to calculate the correlation between cell content and gene expression in all spots and visualized.

The anti-cancer immune status within the tumor immune cycle comprehensively reflects the diverse activities inherent in cancer immune responses. The Tracking Tumor Immunophenotype (TIP) database (http://biocc.hrbmu.edu.cn/TIP/) was utilized to assess the correlation between FCN1 and the anti-cancer immune status across various stages of the tumor immune cycle. Furthermore, the Cancer Immunome Atlas (TCIA) database (https://tcia.at/home) was employed to investigate the response of FCN1 high and low expression groups to PD1 and CTLA4 immunotherapies. Additionally, the TIDE database provided data on the correlation between FCN1 and Cytotoxic T Lymphocytes (CTL), as well as KM survival curves in the context of immunotherapy datasets. The Easier tool, designed for predicting biomarker-based immunotherapies using cancer-specific immune response models, facilitated the evaluation of cytolytic activity (CYT), tertiary lymphoid structure signature (TLS), interferon-γ signature (IFNy), T cell-inflamed signature, and chemokine signature (Lapuente-Santana et al., 2021).

### 2.5 Tumor stemness and drug sensitivity analysis

The tumor stemness score derived from RNA expression (RNAss) and the epigenetically regulated RNA expression-based stemness score (EREG.EXPss) were obtained from a prior investigation (Malta et al., 2018). The mRNA-based stemness index (mRNAsi) was determined utilizing the one-class logistic regression machine learning algorithm (OCLR) (Lian et al., 2019). We integrated stemness index and gene expression data of AML samples and evaluated the association between FCN1 and stemness score. Estimation of IC50 values for 198 compounds in GDSC and their correlation with FCN1 expression were conducted using the R package “oncoPredict” (Maeser et al., 2021). Furthermore, the correlation between FCN1 gene expression and drug sensitivity was assessed utilizing samples from the CellMiner database (Reinhold et al., 2012).

### 2.6 Cell culture and quantitative real-time PCR (qRT-PCR)

U937 cells and NB4 cells were obtained from the Chinese Academy of Sciences’ Cell Bank in Shanghai, China. The cells were grown in Roswell Park Memorial Institute (RPMI)-1640 medium (Gibco, USA) containing 10% fetal bovine serum (Vazyme, China), 100 mg/mL streptomycin and 100 U/mL penicillin. Cells were incubated in a humidified atmosphere with 5% CO_2_ at 37°C.

Total RNA was isolated using the TRIzol reagent (Invitrogen, USA), as directed by the manufacturer. 1µg of RNA was used for cDNA synthesis by the Hiscipt III first strand cDNA synthesis kit (Vazyme, China). GAPDH was amplified from each sample to ensure equal cDNA input. Each PCR reaction contained 1 μL of cDNA, 0.6 μL of forward and reverse primers (10 μM), 7.5 μL of ChamQ universal SYBR qPCR Master Mix (Vazyme, China), and 6.3 μL of ddH2O. The PCR reaction parameters were as follows: 10 min of pre-denaturation at 95°C, 40 cycles of denaturation for 15 s at 95°C, 1 min of annealing at 62°C, and 15 s of extension at 72°C. A reaction was required at 60°C for 1 minute and 95°C for 15 seconds in the last extension phase. The forward and reverse primers for *GAPDH* were GGA​GCG​AGA​TCC​CTC​CAA​AAT and GGC​TGT​TGT​CAT​ACT​TCT​CAT​GG, respectively. The forward and reverse primers for *FCN1* were GGC​AGG​TGT​CAT​TGG​AGA​GAG and GTC​GCA​CAC​GAC​TGA​GAC​TG, respectively.

### 2.7 Western blotting

Cells were harvested following treatment with siRNAs. The cells were then collected by centrifugation after being cleaned three times with phosphate-buffered saline (PBS). Proteinase inhibitors (Solarbio, China) were added to RIPA buffer to help create total protein extracts. *FCN1* (Proteintech, China) and *GAPDH* (Proteintech, China) antibodies were used in line with the manufacturer’s instructions for western blot analysis. Goat Anti-Mouse IgG-HRP (Proteintech, China) and Goat Anti-Rabbit IgG-HRP (Proteintech, China) were the secondary antibodies used. The protein loading control was *GAPDH*. The enhanced chemiluminescence (ECL) reagent (4A Biotech, China) was used to visualize the signals. SiRNA1 sense: CCG​ACU​GUC​AUG​CUU​CAA​A, antisense: UUU​GAA​GCA​UGA​CAG​UCG​G. SiRNA2 sense: GCU​AGU​CUU​GUU​CCU​GCA​U, antisense: AUG​CAG​GAA​CAA​GAC​UAG​C.

### 2.8 Cell viability assay

Cell viability was evaluated using the Cell Counting Kit-8 (CCK-8) (APExBIO, USA). U937 cells and NB4 cells transfected with siRNA2-*FCN1* were harvested upon reaching 60% confluency. They were then seeded onto 96-well culture plates, with five multiple wells allocated to each group, and 5,000 cells per well. The CCK-8 kit was used to examine the cells at 0 h, 24 h, 48 h, and 72 h after they were incubated in an incubator with 37°C and 5% CO_2_.

### 2.9 Flow cytometric analysis of apoptosis and cell cycle

After being harvested, U937 cells and NB4 cells were suspended in a binding buffer. The cells were then stained in accordance with the manufacturer’s instructions using the Annexin V-APC/PI Apoptosis Detection Kit (KeyGen Biotech, China). A Beckman Cytoflex device was used for the flow cytometry analysis, and CytExpert Software was used to analyze the data.

The cell cycle of U937 cells and NB4 cells were detected by Cell Cycle Detection Kit (KeyGen Biotech, China). In brief, cells were collected and fixed in 70% cold ethanol overnight at 4°C. After washing with PBS twice, cells were incubated with PI/RNase a staining buffer for 30 min and subsequently analysed by Beckman flow cytometry and CytExpert Software.

### 2.10 Statistical analysis

In addition to online databases, R version 4.2.1 was employed for all statistical analyses. Unpaired Wilcoxon Rank Sum and Signed Rank Tests were utilized to assess the significance of differences between pairs, while kruskal. test was employed for testing differences among multiple groups of samples. Survival analysis was conducted using KM curves, accompanied by log-rank tests or Cox proportional hazards regression models. Pearson or Spearman correlation coefficients were employed to evaluate variable relationships. All cellular experiments were conducted in triplicate, and results are presented as mean ± standard deviation. Cell experiment data analysis was performed using GraphPad Prism for Windows (version 9.0.0). A significance level of *p* < 0.05 was defined as statistically significant (*p < 0.05, **p < 0.01, ***p < 0.001; ns: not significant).

## 3 Results

### 3.1 Pan-cancer expression landscape of FCN1

**FIGURE 1:**
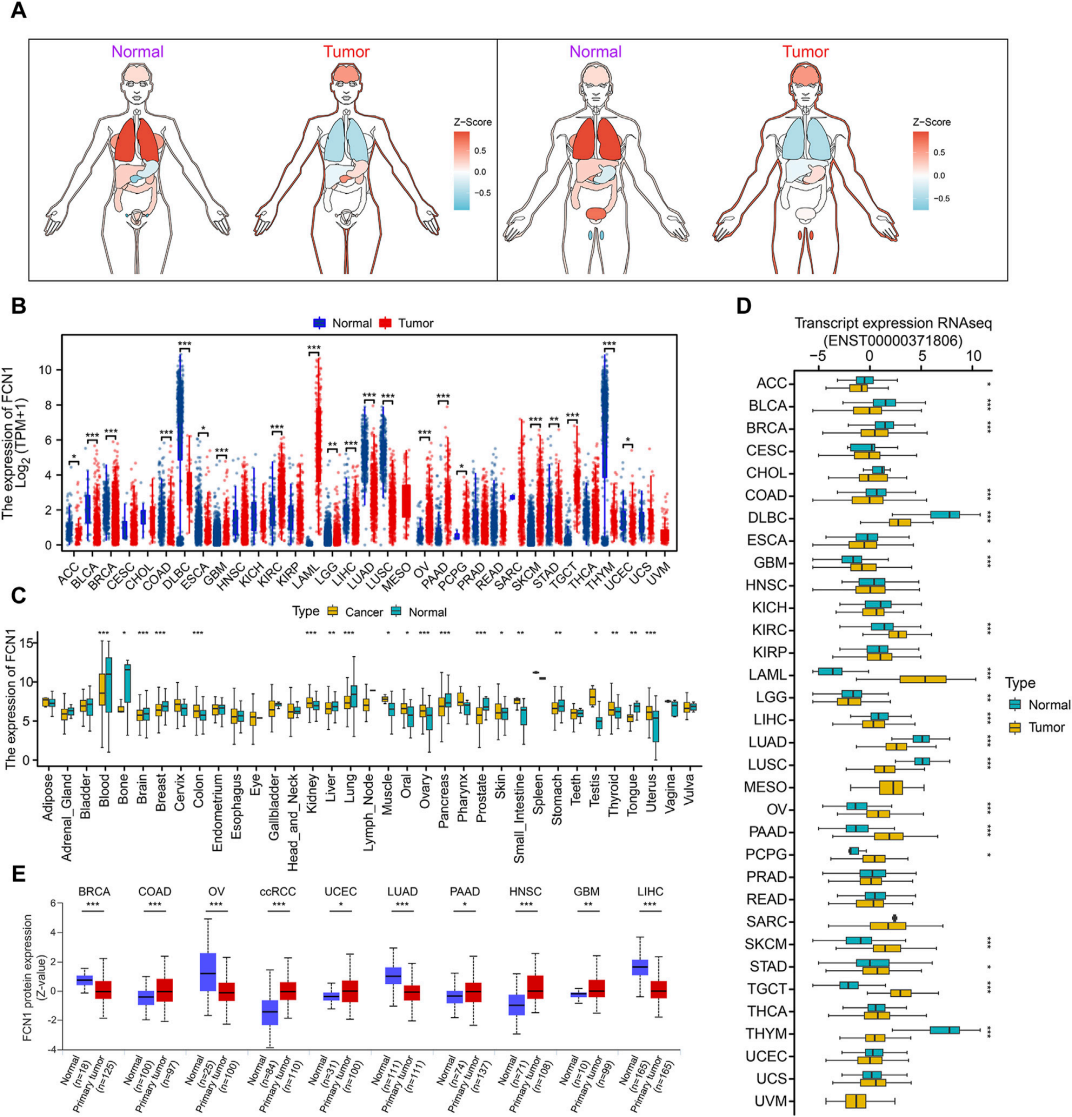
**(A)** TCGA combined with GTEx analysis of pan-cancer FCN1 expression organogram; **(B)** Differential expression of FCN1 in normal tissues and tumor tissues (TCGA + GTEx); **(C)** GENT2 database analyzes differential expression of FCN1 between normal and tumor samples across a wide range of tissues; **(D)** Differential expression of transcripts encoding FCN1 protein between pan-cancer normal and tumor tissues; **(E)** UALCAN analyzes FCN1 protein expression levels between pan-cancer normal and tumor samples.

The expression of FCN1 in normal human tissues exhibits high specificity, with FCN1 mRNA predominantly expressed in bone marrow and lymphoid tissues (Supplementary Figure S1A). Cancer cell line profiling revealed FCN1 expression in cancer cell lines (Supplementary Figure S1B). Assessment of FCN1 expression across 33 tumor datasets from TCGA revealed dysregulated expression in over one-third of tumor types compared to normal tissue (Supplementary Figure S1C). Paired sample analysis demonstrated low FCN1 expression in multiple tumor samples, such as bladder urothelial carcinoma (BLCA), breast invasive carcinoma (BRCA), and colon adenocarcinoma (COAD), while it exhibited high expression in clear renal cell carcinoma (KIRC) (Supplementary Figure S1D). Integration of samples from TCGA and GTEx unveiled dysregulated FCN1 expression in 21 of 33 cancer types, particularly prominent in AML (Figures 1A, B). Additionally, we corroborated FCN1 expression imbalances using the GENT2 database (Figure 1C). Furthermore, we observed unbalanced expression of the FCN1 protein transcript in 20 cancers, with significantly elevated expression in AML and markedly reduced expression in most other cancers (Figure 1D). Protein-level analysis revealed significantly decreased FCN1 levels in BRCA, Ovarian serous cystadenocarcinoma (OV), Lung adenocarcinoma (LUAD), and Liver hepatocellular carcinoma (LIHC), whereas it was significantly elevated in COAD, clear cell renal cell carcinoma (ccRCC), Uterine Corpus Endometrial Carcinoma (UCEC), Pancreatic adenocarcinoma (PAAD), Head and Neck squamous cell carcinoma (HNSC), and Glioblastoma multiforme (GBM) (Figure 1E). Furthermore, FCN1 expression exhibited significant associations with molecular subtypes and clinical stages across various tumors (Supplementary Figure S1E; Supplementary Figure S2A), suggesting that dysregulated FCN1 expression in diverse cancers may contribute to tumorigenesis and progression.

### 3.2 Mutations, epigenetic alterations, and genomic heterogeneity of FCN1 in pan-cancer

The mutation rate of FCN1 varies across different cancer types, with notably higher rates observed in UCEC and Skin Cutaneous Melanoma (SKCM) (Supplementary Figure S3A, B). Furthermore, Missense Mutations are predominant in FCN1 alterations. Copy number variation (CNV) analysis reveals an increased frequency of amplifications in Adrenocortical Carcinoma (ACC) and HNSC, while deletions are more prevalent in Cholangiocarcinoma (CHOL), OV, Uterine Carcinosarcoma (UCS), and other cancers (Supplementary Figure S3C). Genomic heterogeneity analysis reveals significant positive correlations between FCN1 and MSI and Mutant-Allele Tumor Heterogeneity (MATH) in AML, contrasting with negative correlations observed in TGCT (Supplementary Figure S3D, E). HRD status, a critical indicator for treatment selection and prognosis in various tumors, demonstrates significant positive correlations with FCN1 in Basal-like BRCA and OV, while displaying negative associations with Mesothelioma (MESO) and Lung Squamous Cell Carcinoma (LUSC) (Supplementary Figure S3F). Furthermore, FCN1 exhibits significant positive correlations with LOH in BRCA and negative associations with DLBC, KIRC, and MESO (Supplementary Figure S3G). Collectively, these findings underscore a pivotal relationship between FCN1 and genomic instability across diverse cancer types. Additionally, we conducted a comprehensive examination of FCN1’s epigenetic modifications. Supplementary Figure S3H illustrates the methylation probe levels of FCN1 across various cancers. Differential analysis highlights a significant decrease in FCN1 methylation levels in BLCA, HNSC, LIHC, and other cancers, particularly notable at Transcription Start Sites (TSS) (Supplementary Figure S3I). Correlation analysis reveals a significant positive relationship between FCN1 mRNA expression and methylation across multiple cancers (Supplementary Image 4A). Moreover, this correlation varies across cancer types, with positive associations observed in BLCA, COAD, and SKCM, and negative correlations in AML, Testicular Germ Cell Tumors (TGCT), and Thymoma (THYM) (Supplementary Figure S4A-E). Survival analysis indicates that hypermethylation of FCN1 is significantly associated with longer OS in Lymphoid Neoplasm Diffuse Large B-cell Lymphoma (DLBC), GBM, Kidney Renal Papillary Cell Carcinoma (KIRP), Uveal Melanoma (UVM), and other cancers (Supplementary Figure S4F-I). Additionally, FCN1 methylation shows significant positive correlations with Cytotoxic T Lymphocytes (CTL) in Basal-like BRCA, HNSC, and Melanoma, while displaying negative associations in AML (Supplementary Figure S4J-M).

### 3.3 FCN1 linked to inflammation and immune response in cancer

**FIGURE 2:**
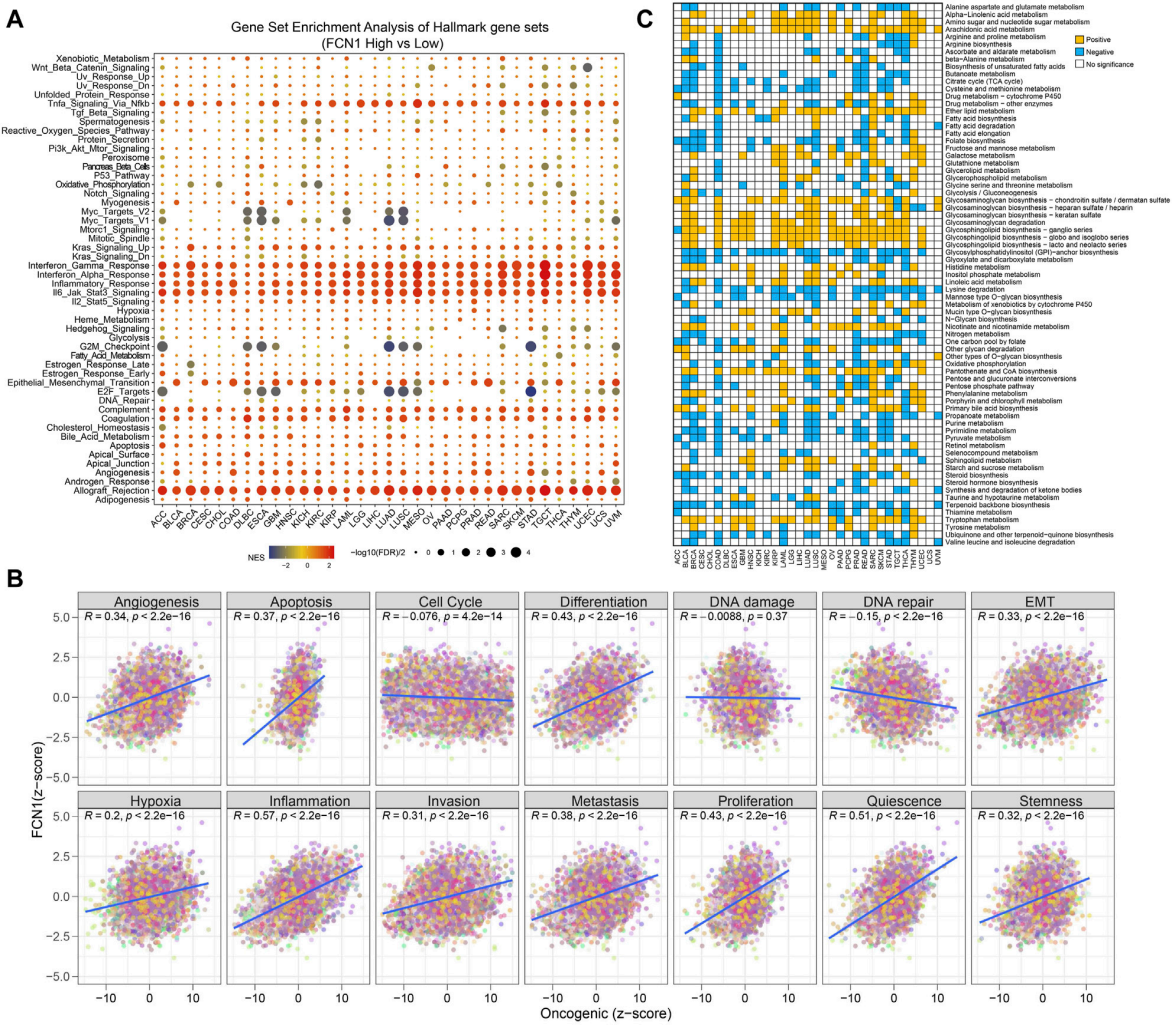
**(A)** Potential function analysis of FCN1 in human cancers using GSEA.; **(B)** Pearson correlation between FCN1 and 14 tumor states collected by CancerSEA database; **(C)** Differences in metabolic pathway GSVA scores between FCN1 high-expression group and low-expression group in pan-cancer.

Gene set enrichment analysis reveals significant associations between FCN1 and several pathways in pan-cancer analysis, including INFLAMMATORY_RESPONSE, INTERFERON_ALPHA_RESPONSE, INTERFERON_GAMMA_RESPONSE, ALLOGRAFT_REJECTION, and TNFA_SIGNALING_VIA_NFKB (Figure 2A). Conversely, certain cancers exhibit a notable negative correlation with pathways such as MYC_TARGETS_V2, MYC_TARGETS_V1, and G2M_CHECKPOINT. CancerSEA data further corroborates these findings, demonstrating significant relationships between FCN1 and pan-cancer pathways such as Angiogenesis, Apoptosis, Differentiation, Epithelial-Mesenchymal Transition (EMT), Inflammation, and Metastasis (Figure 2B). Moreover, metabolic pathways, including Glycosphingolipid biosynthesis and degradation, Tryptophan metabolism, and Arachidonic acid metabolism, are activated in the FCN1 high-expression group across multiple cancers such as BRCA, COAD, AML, and LUAD. Conversely, pathways such as Lysine degradation and the Citrate cycle (TCA cycle) are inhibited in the FCN1 high-expression group in select cancers (Figure 2C; Supplementary Figure S5A, D).

### 3.4 The immunological landscape of FCN1 in pan-cancer

**FIGURE 3:**
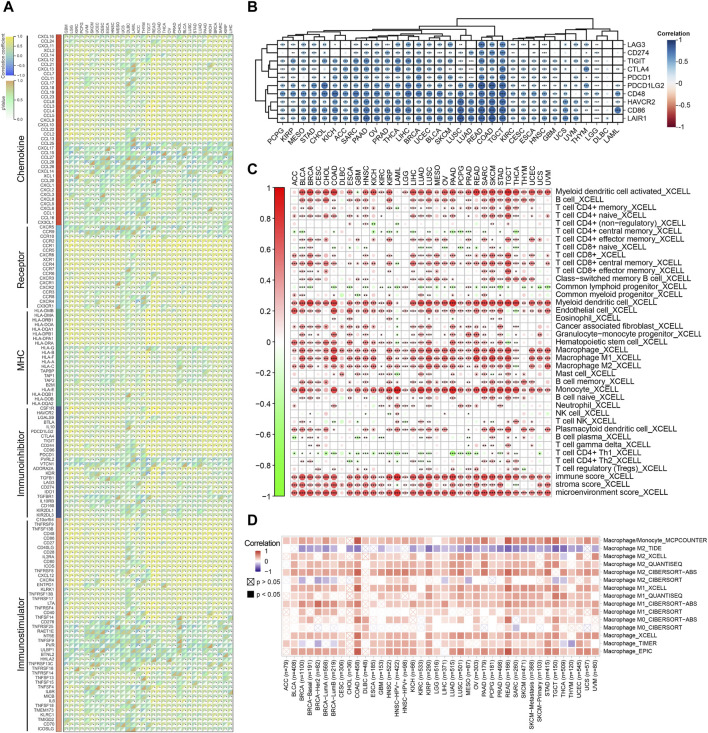
**(A)** Correlation between FCN1 and 150 immune regulatory factors in pan-cancer; **(B)** Correlation between FCN1 and immune checkpoints in pan-cancer; **(C)** XCELL algorithm evaluates the correlation between FCN1 and immune cell infiltration; **(D)** TIMER2.0 database evaluates the correlation between FCN1 and macrophage infiltration.

**FIGURE 4:**
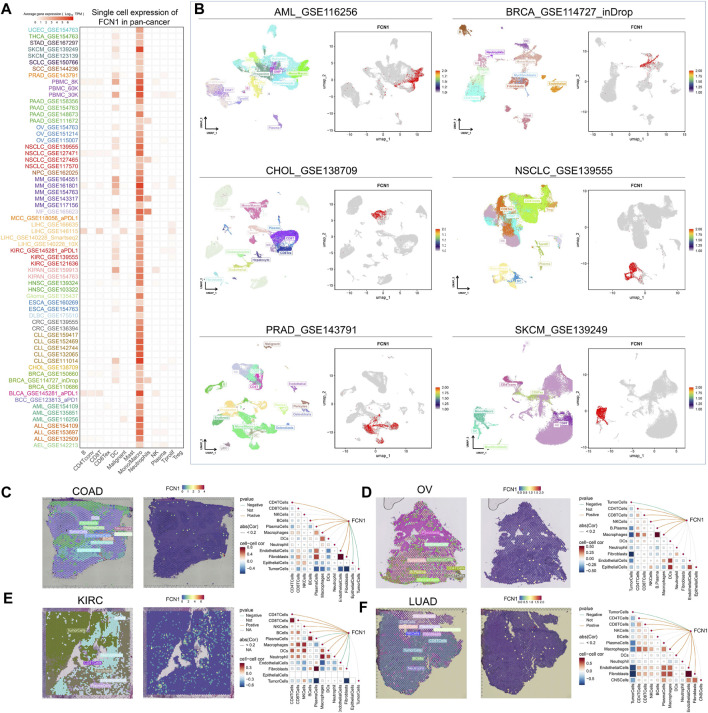
**(A)** Single cell expression of FCN1 in pan-cancer (TISCH2 database); **(B)** UMAP maps of single cell data sets from AML, BRCA, CHOL, NSCLC, PRAD, SKCM and single cell localization maps of FCN1; **(C–F)** From left to right: Cell positioning after spatial transcriptome deconvolution, the maximum value of each spot cell component, FCN1 spatial transcriptome positioning, spearman correlation between FCN1 expression and microenvironmental components at idle resolution.

Initially, we found that FCN1 was significantly associated with multiple immune subtypes of cancer (Supplementary Figure S6). We conducted an analysis of the correlation between FCN1 and 150 immune regulatory factors encompassing chemokines (41), receptors (18), MHC molecules (21), immunoinhibitors (24), and immunostimulators (46) across various cancer types. Notably, several inflammatory chemokines, such as CCL2, CCL3, and CCL5, exhibited significant upregulation in the FCN1 high-expression cohort, thereby facilitating the phagocytic activities of monocytes and macrophages as well as inducing inflammatory responses within tissues. Moreover, MHC molecules, including HLA-DOA, were markedly upregulated in the FCN1 high-expression group, indicative of enhanced antigen presentation and processing capabilities within this subset. Concurrently, immunostimulators such as CD48 were also found to be elevated in the FCN1 high-expression cohort (Figure 3A). Additionally, several common immune checkpoint molecules, such as CD86, TIGIT, CTLA4, and PDCD1, exhibited significant positive correlations with FCN1 (Figure 3B). Subsequent immune infiltration analyses revealed a substantial positive correlation between FCN1 expression and immune cell infiltration, including immune score, myeloid dendritic cells, monocytes, macrophages, CD8^+^ central memory T cells, and cancer-associated fibroblasts, while demonstrating a significant negative correlation with common lymphoid progenitors (Figure 3C; Supplementary Figure S7A). Furthermore, validation of these findings across multiple algorithms and datasets, including the TIMER2.0 database, provided additional support (Figure 3D; Supplementary Figure S7G). Single-cell transcriptional data sourced from TISCH corroborated the expression of FCN1 primarily in macrophages across various cancer types (Figure 4A). Notably, FCN1 exhibited significantly elevated expression levels in macrophages across several cancers, including AML, BRCA, CHOL, NSCLC, PRAD, and SKCM (Figure 4B). Additionally, the comparison of cell type proportions between FCN1-positive and -negative groups revealed a higher abundance of macrophages in the FCN1-positive cohort (Supplementary Figure S8A). Pathway analysis conducted on the AML_GSE116256 dataset unveiled distinct pathway differences between FCN1-positive and -negative cell types, with immune and signaling pathways exhibiting higher scores in the FCN1-positive group, while proliferation-related pathways scored higher in the FCN1-negative group (Supplementary Figure S8G). Lastly, analysis of four spatial transcriptome datasets for COAD (CRC_WholeTranscriptomeAnalysis_10x), OV (GSE203612-GSM6177614), KIRC (GSE175540-GSM5924030_ffpe_c_2), and LUAD (BrainMetastasis_GSE179572-GSM5420754) revealed a significant albeit not overwhelmingly strong correlation between FCN1 expression and macrophages in COAD, OV, KIRC, and LUAD (Figures 4C–F). Furthermore, comparative analysis of FCN1 expression levels in malignant cells, mixed malignant cells, and non-malignant cells unveiled significantly higher expression in non-malignant cells (Supplementary Figure S8H).

**FIGURE 5:**
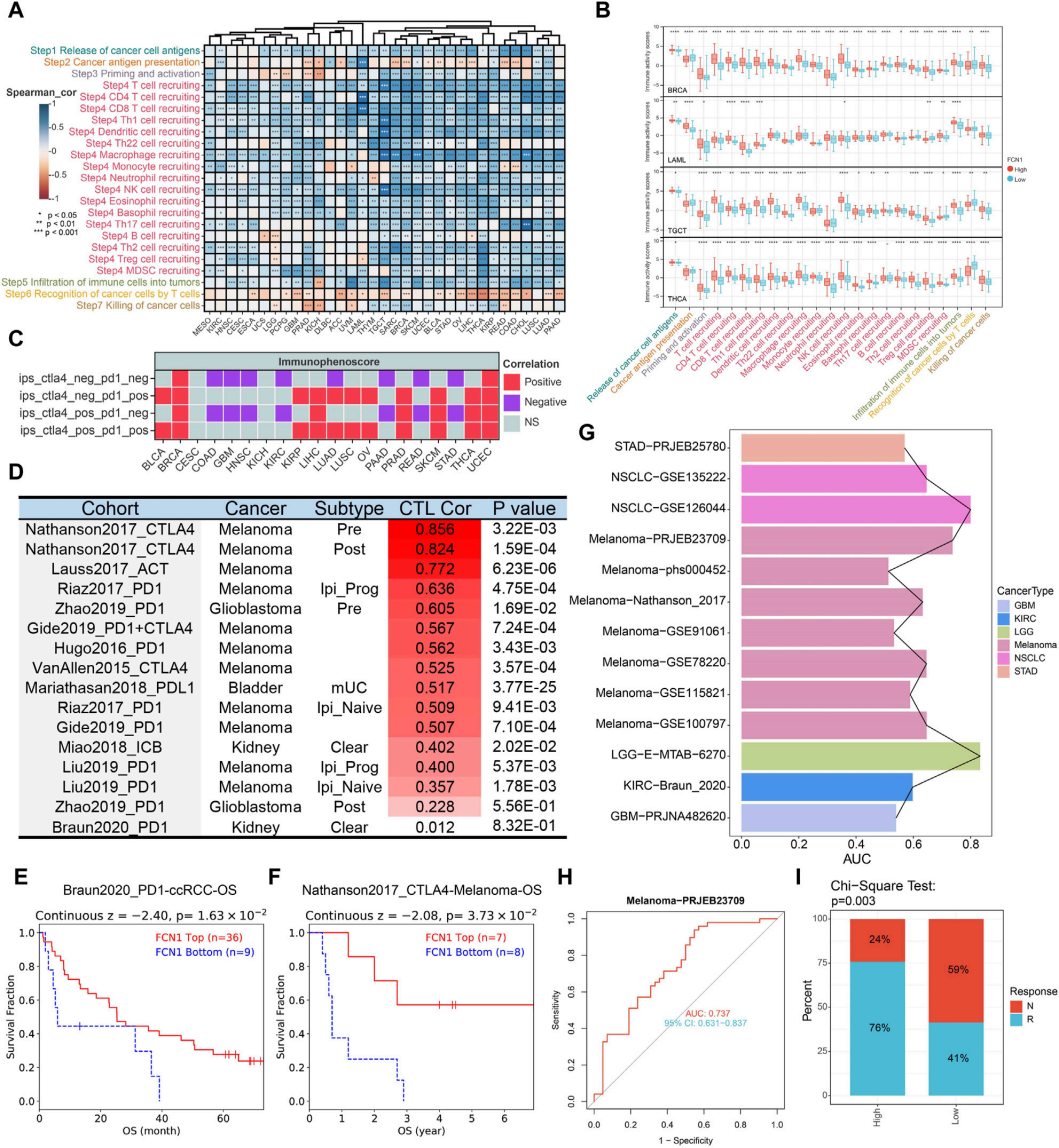
**(A)** Heat map showing correlation between FCN11 expression and the cancer immune cycle in pan-cancer; **(B)** Representative boxplots display the correlation analysis of the cancer-immunity cycle in BRCA, LAML, TGCT, and THCA; **(C)** TCIA database evaluates association between FCN1 and immunotherapy; **(D)** TIDE database evaluates the correlation between FCN1 and CTL in immunotherapy datasets; **(E)** KM curves evaluated the association between FCN1 expression and OS of PD1-treated ccRCC; **(F)** KM curves evaluated the association between FCN1 expression and OS of CTLA4-treated Melanoma; **(G)** Pan-cancer FCN1 expression predicts ROC-AUC values in immunotherapy response and non-responder patients; **(H)** FCN1 expression predicts immunotherapy response and non-responder patients ROC curve (Melanoma-PRJEB23709 data set); **(I)** Chi-square test detects the significance of the difference in the proportion of patients with immunotherapy response and non-response between the FCN1 high expression group and the low expression group (Melanoma-PRJEB23709 data set).

The immune status against cancer within the tumor immune cycle inherently mirrors the diverse activities encompassed in the cancer immune response (Chen and Mellman, 2017). Our investigation delved into the relationship between FCN1 and various stages of the cancer immune cycle. Apart from the notable inverse association observed with T cell recognition of cancer cells (Step 6), FCN1 exhibited a significant positive correlation with other stages, notably the recruitment of multiple immune cells during immune cell trafficking towards tumors (Step 4) (Figures 5A, B). Leveraging TCIA data, we assessed FCN1’s responsiveness to immunotherapy. Notably, FCN1 displayed a significant positive correlation with ips_ctla4_neg_pd1_pos and ips_ctla4_pos_pd1_pos across diverse cancer types (Figure 5C; Supplementary Figure S9). Furthermore, noteworthy positive correlations between FCN1 and CTL were observed across multiple immunotherapy datasets (Figure 5D). Survival analysis underscored a significant association between high FCN1 expression and prolonged OS in PD1-treated ccRCC and CTLA4-treated melanoma cohorts (Figures 5E, F). Additionally, we scrutinized the predictive capacity of FCN1 in gauging immunotherapy response. Notably, FCN1 exhibited variable prediction performance across datasets, with superior performance observed in the melanoma dataset PRJEB23709 (AUC: 0.737; 95%CI: 0.631–0.837) (Figures 5G, H). Moreover, a higher proportion of patients in the FCN1 high expression group within the melanoma dataset PRJEB23709 demonstrated responsiveness to immunotherapy (Figure 5I).

### 3.5 FCN1 offers diagnostic and prognostic Utility in different cancers

Through univariate Cox and log-rank test analyses, we discerned that FCN1 exhibited prognostic significance across a spectrum of cancers, excluding BRCA, KICH, KIRP, Pheochromocytoma and Paraganglioma (PCPG), Rectum adenocarcinoma (READ), Thyroid carcinoma (THCA), and UCEC (Supplementary Figure S10A). Subsequent scrutiny revealed FCN1’s role as a protective factor in multiple cancers, with heightened FCN1 expression significantly correlating with extended OS and Progression-Free Interval (PFI) in ACC, CHOL, HNSC, LIHC, and SKCM. Conversely, heightened FCN1 expression correlated significantly with abbreviated OS in COAD, GBM, KIRC, LUSC, STAD, TGCT, and THYM. Notably, FCN1 also displayed varied associations with DSS and DFI across multiple cancer types (Supplementary Figures S10B, C). Additionally, ROC curves hinted at FCN1’s potential as a diagnostic biomarker in select cancers (Supplementary Figure S11A-L).

**FIGURE 6:**
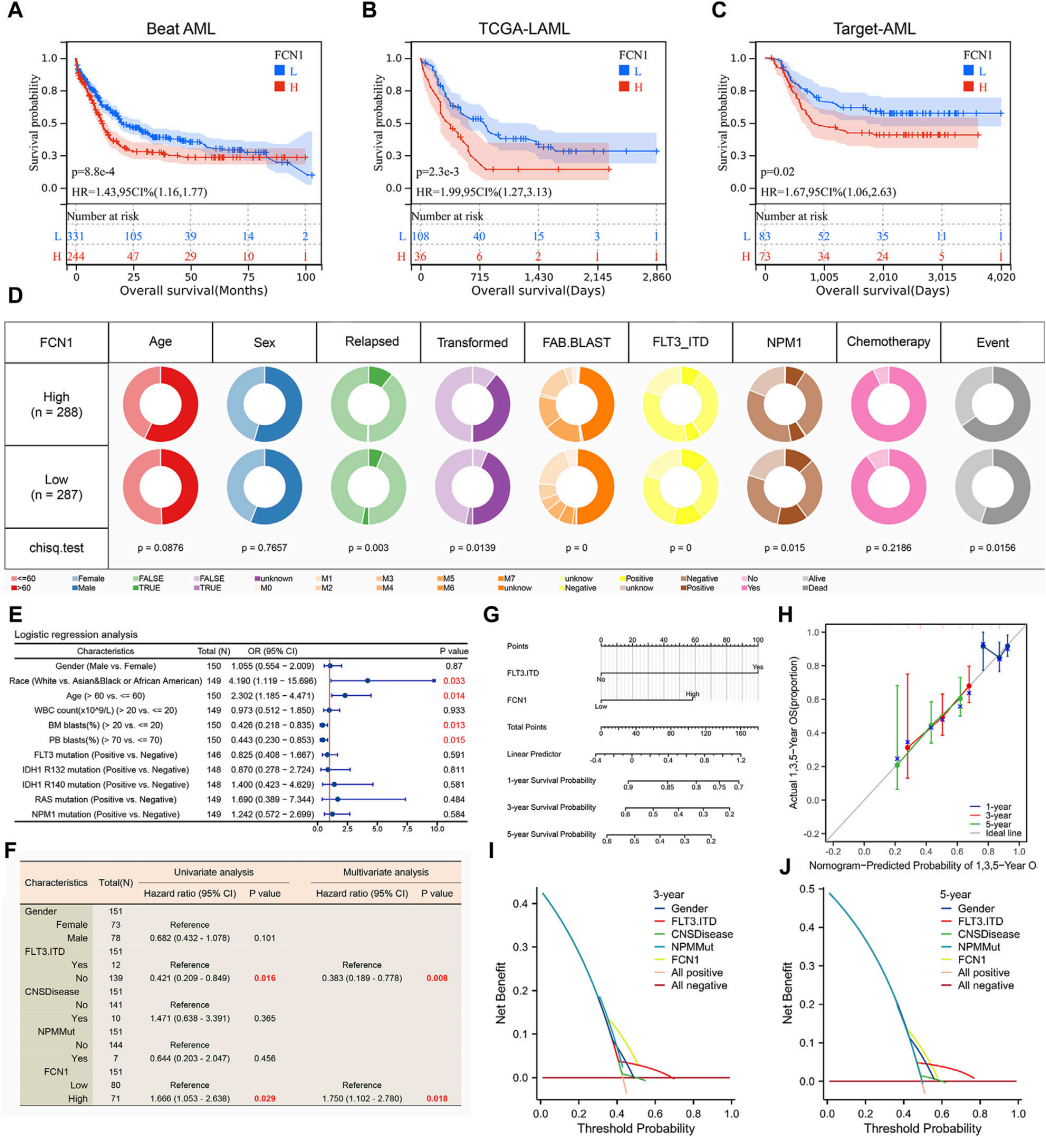
**(A–C)** High expression of FCN1 is significantly associated with shorter OS in AML patients; **(D)** Correlation between FCN1 and clinical features of AML (Beat AML cohort); **(E)** Logistic regression analysis of the correlation between FCN1 and clinical characteristics (TCGA-LAML cohort); **(F)** Univariate and multivariate cox regression analysis to evaluate the independent prognostic value of FCN1 (Target-AML cohort); **(G)** Nomogram constructed based on FCN1 and clinical factors; **(H)** Calibration curves evaluate prognostic model accuracy at 1, 3, and 5 years; **(I, J)** The DCA curve evaluates the predictive value of the model for 3 and 5 years.

Given the aberrant expression and dysregulation of FCN1 in leukemia, our investigation primarily focused on elucidating its association with clinical characteristics and prognosis in AML. Through meticulous examination across six datasets containing survival data of AML patients, we consistently observed a significant correlation between elevated FCN1 expression and reduced OS in AML cohorts (Figures 6A–C; Supplementary Figure S12A-C). Furthermore, we meticulously analyzed FCN1’s relationship with various clinical features of AML. In the Beat AML dataset, FCN1 exhibited notable associations with clinical relapse, disease transformation, FAB blast subtype, FLT3-ITD mutations, and NPM1 mutations (Figure 6D; Supplementary Figure S12D). Employing logistic regression analysis on the TCGA-LAML dataset, FCN1 emerged significantly correlated with Race, Age, Bone Marrow (BM) blasts, and Peripheral Blood (PB) blasts (Figure 6E; Supplementary Figure S12I). Subsequent univariate and multivariate Cox regression analyses on the Target-AML dataset underscored the robust association between heightened FCN1 expression and diminished OS in AML patients, establishing FCN1 as an independent prognostic indicator (Figure 6F). To facilitate clinical prognostication, we developed a nomogram predicting one-, three-, and 5-year survival probabilities for AML patients (Figure 6G). Calibration curves and decision curve analysis further validated the efficacy of FCN1 in prognosticating three- and 5-year survival outcomes in AML (Figures 6H–J).

### 3.6 Functional enrichment analysis of FCN1 in AML

We investigated the correlation between effector genes of tumor-associated immune cells (CD8 T-cell, NK cell, macrophage, Th1 cell, Dendritic cell) and FCN1 expression. Our analysis revealed a significant and positive correlation between multiple effector genes of macrophages and dendritic cells with FCN1 expression (Supplementary Figure S13A). Considering the substantial correlation observed between FCN1 and immune inflammation, we further assessed the relationship between FCN1 and various immune-inflammatory gene sets, which demonstrated a positive correlation (Supplementary Figure S13B). Utilizing EaSIeR, we evaluated five scores indicative of response to immune checkpoint blockade (ICB) treatment and found significant positive correlations between FCN1 and CYT, TLS, IFNy, Tcell_inflamed, and chemokines in AML (Supplementary Figure S13G). Moreover, we observed that the FCN1 high expression group in AML exhibited lower TIDE scores, suggesting potential benefits from ICB treatment (Supplementary Figure S13H). These findings underscore the critical role of FCN1 in modulating the immune microenvironment of AML.

At the genetic level, FCN1 exhibited interactions with FCN2, FFAR2, and MASP2 proteins (Supplementary Figure S14A). Moreover, FCN1 demonstrated interactions with FCN2, FCN3, and COLEC11 proteins (Supplementary Figure S14B). Through a comprehensive analysis of the FCN1 high- and low-expression groups within the LAML dataset, we identified 773 upregulated genes and 349 downregulated genes (Supplementary Figure S14C). Spearman correlation analysis revealed 559 genes exhibiting significant positive correlation with FCN1, alongside 15 genes displaying significant negative correlation (Supplementary Figure S14D). Integration of these findings delineated 391 overlapping genes, which were designated as pivotal in FCN1 functionality (Supplementary Figure S14E). Functional enrichment analysis, focusing on biological processes, highlighted the enrichment of hub genes in pathways associated with positive regulation of cytokine production, immune response-regulating signaling pathways, among others. Additionally, cell component annotation revealed enrichment of differential genes in pathways inclusive of secretory granule membranes, while molecular function annotation indicated enrichment in pathways involving immune receptor activity, among others (Supplementary Figure S14F). KEGG analysis further elucidated the enrichment of differential genes in various immune and cancer-related pathways, including neutrophil extracellular trap formation, cytokine-cytokine receptor interaction, and apoptosis (Supplementary Figure S14G).

### 3.7 Correlation between FCN1 and AML stemness score and drug sensitivity

**FIGURE 7:**
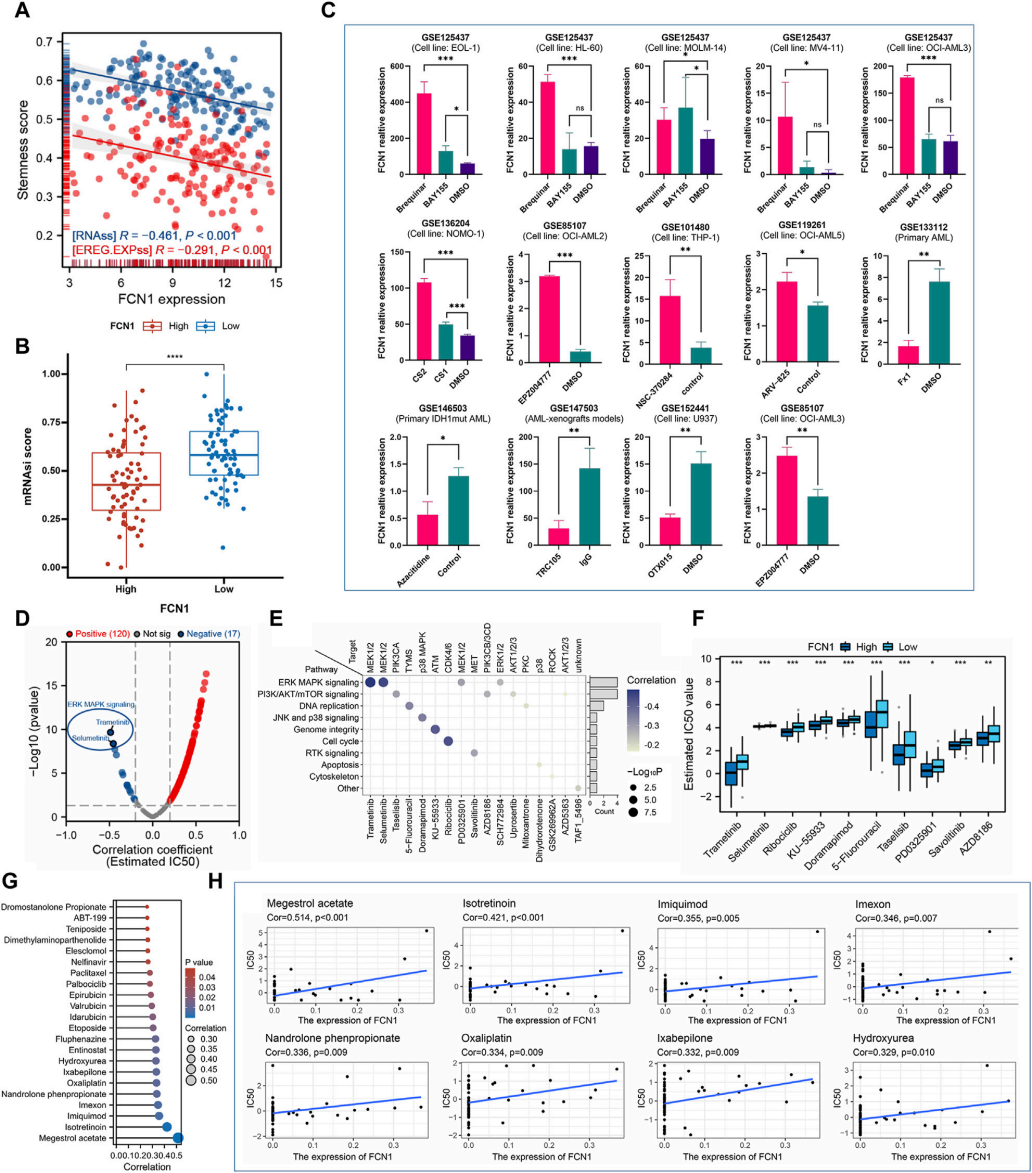
**(A)** FCN1 is significantly negatively correlated with RNAss and EREG. EXPss in AML; **(B)** The FCN1 low expression group in AML had a higher stemness score; **(C)** Multiple GEO datasets assess dysregulation of FCN1 expression in drug-treated and control groups; **(D)** Evaluating the correlation between FCN1 and 19 drugs based on GDSC2; **(E)** Signaling pathways and target genes corresponding to 17 drugs with significant negative correlations; **(F)** Difference IC50 of the top 10 drugs in FCN1 high and low expression groups; **(G)** Evaluation of the relationship between FCN1 and drug sensitivity based on Cellminer; **(H)** Scatter plot of correlation between top drugs and FCN1.

The stemness score is intricately linked to the emergence of drug resistance and sustained proliferation in tumor cells during the treatment of malignant tumors. Prior research suggests that the maintenance of AML is mediated by rare leukemia stem cells (LSCs) (Ma et al., 2021; Gudmundsson and Du, 2023). Our analysis revealed a significant negative correlation between FCN1 and RNAss and EREG. EXPss in AML (Figure 7A). Notably, the FCN1 low expression group exhibited a higher tumor stemness score (Figure 7B). Elevated FCN1 expression potentially suppresses the stemness characteristics of AML cells, indicating that patients in the low FCN1 expression group might display heightened resistance to traditional anticancer therapies. To further elucidate the relationship between FCN1 and drug sensitivity, we curated multiple datasets on leukemia drug treatments from literature sources. FCN1 exhibited significant differential expression across various drug treatment groups, including Brequinar, BAY155, CS2, CS1, EPZ004777, and Azacitidine, compared to the control group (Figure 7C). We calculated the IC50 values of 198 compounds from the GDSC database for each AML sample and conducted Spearman correlation analysis, revealing 120 drugs positively correlated with FCN1 and 17 negatively correlated drugs. Notably, Trametinib and Selumetinib, inhibitors of the ERK MAPK signaling pathway, displayed the largest negative correlation coefficients (Figure 7D). We synthesized the signaling pathways and therapeutic targets of the 17 identified drug candidates (Figure 7E). Furthermore, a boxplot illustrated the IC50 values of the top 10 drugs in both FCN1 high- and low-expression groups (Figure 7F). Leveraging the Cellminer dataset, we assessed the correlation between FCN1 gene expression and drug activity, revealing significant positive associations with multiple drugs (Figure 7G). Notably, the top eight drugs included megestrol acetate, isotretinoin, imiquimod, imexon, nandrolone phenpropionate, oxaliplatin, ixabepilone, and hydroxyurea (Figure 7H).

### 3.8 FCN1 regulates the proliferation and apoptosis of AML cells

**FIGURE 8:**
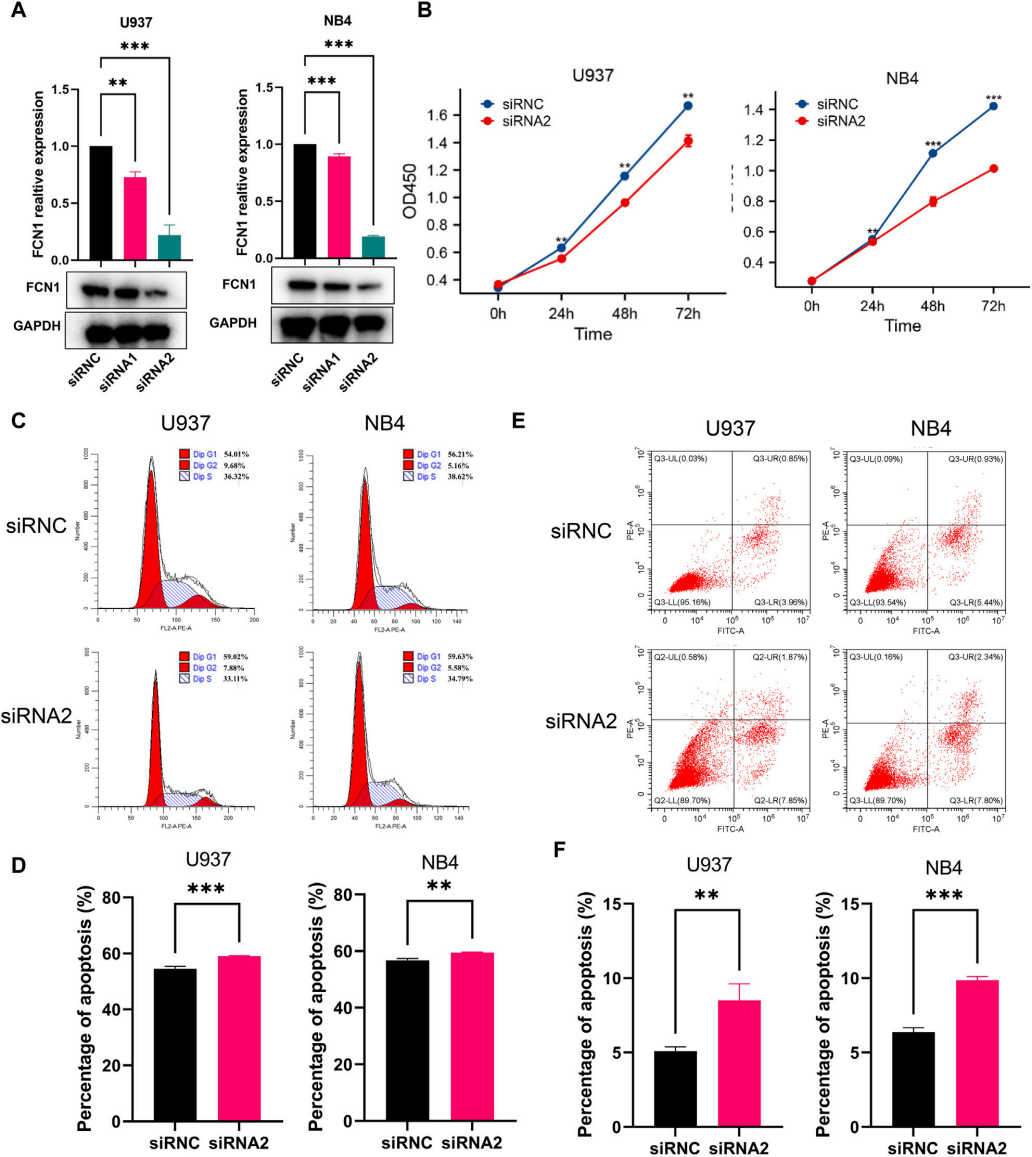
**(A)** Verification of the knockout efficiency of FCN1 in U937 cells and NB4 cells by RT-PCR and Western blot; **(B)** CCK8 experiment to analyze the effect of knocking out FCN1 on cell proliferation; **(C)** Flow cytometric analysis of the cell cycle changes of U937 and NB4 cell lines after FCN1 knockdown; **(D)** The percentage of G0/G1 phages in U937 cells and NB4 cells after knockdown of FCN1; **(E)** Flow cytometric analysis of changes in apoptosis in U937 and NB4 cell lines after FCN1 knockdown; **(F)** The apoptosis rates of U937 cells and NB4 cells after knockdown of FCN1.

U937 cells and NB4 cells underwent transfection with two distinct siRNA constructs, followed by RT-PCR and western blot analyses. The mRNA expression levels were markedly reduced in the transfected group compared to the control, but protein expression levels was only markedly reduced in the siRNA2 transfected group compared to the control (Figure 8A). Consequently, siRNA2 was chosen for subsequent investigations. Notably, the CCK-8 assay revealed a significant inhibition of cell proliferation upon FCN1 deficiency in U937 cells and NB4 cells, in contrast to cells transfected with an empty vector (siRNC) (Figure 8B). Moreover, the proportion of cells in the G0/G1 phase substantially increased in the FCN1-knockdown group, indicative of cell cycle arrest and proliferation suppression (Figures 8C, D). Intriguingly, FCN1 knockdown led to a notable rise in the apoptosis rate of U937 cells and NB4 cells (Figures 8E, F), contrary to prior analyses. Consequently, we delved into the correlation between FCN1 and genes associated with apoptosis pathways. Our findings revealed significant positive correlations between FCN1 and several anti-apoptotic genes, including LGALS3, IFNGR1, and MCL1, while displaying significant negative correlations with various pro-apoptotic genes such as CASP6, CASP3, and BIK (Supplementary Figure S14H). This observation rationalizes the observed increase in apoptosis rate upon FCN1 knockdown in U937 cells and NB4 cells, indicating the regulatory role of FCN1 in fundamental biological processes such as proliferation, apoptosis, and cell cycle regulation.

## 4 Discussion

Numerous prior investigations have thoroughly elucidated the significance of FCN1, highlighting its pivotal involvement in inflammatory response and immune defense mechanisms (Vander et al., 2007; Munthe-Fog et al., 2012; Addobbati et al., 2016; Okuzaki et al., 2017; MacDonald et al., 2021; Chen et al., 2023). More recently, attention has been drawn to its potential prognostic implications in hepatocellular carcinoma, endometrial cancer, and gastric adenocarcinoma (Kaur et al., 2021; Khan et al., 2022; Sun et al., 2022). Building upon these foundational studies, the current research endeavors to comprehensively examine the expression patterns, prognostic value, and functional roles of FCN1 across various cancer types, with a particular emphasis on its relevance in AML.

This study presents a pioneering evaluation of FCN1’s distinct expression profiles at mRNA, transcript, and protein levels across 33 tumors, marking the first pan-cancer analysis of its kind. Notably, FCN1 expression demonstrates dysregulation across most tumor types, particularly in AML. Post-translational modifications, such as methylation, hold potential to influence protein stability and consequently affect expression levels (Xu et al., 2021). Discrepancies between FCN1 mRNA and protein expression in COAD, OV, suggest a potential mediation by methylation alterations, known to play pivotal roles in tumor initiation and progression. Nevertheless, rigorous experimental validation is imperative to elucidate the specific regulatory mechanisms involving FCN1 and methylation modifications, thus providing a promising avenue for subsequent research into FCN1’s oncogenic mechanisms. Our findings also unveil FCN1’s dual prognostic role, acting as a protective factor in ACC, LUAD, SARC, and SKCM, while posing as a prognostic risk factor in ESCA, LUSC, STAD, and AML. This dual prognostic behavior may stem from the inherent tumor heterogeneity (Lawson et al., 2018). Sokołowska et al. identified FCN1 as a candidate complementary biomarker for AML based on clinical samples (Sokołowska et al., 2020). Our investigation underscores FCN1’s significant association with various clinical features of AML, establishing FCN1 as an independent prognostic indicator for this malignancy.

The tumor microenvironment and infiltration of immune cells play pivotal roles in tumor development and the response to immunotherapy (Xiao and Yu, 2021). FCN1 likely participates in regulating the tumor microenvironment, influencing tumor initiation and progression. Monocytes and macrophages are crucial constituents of the immune system, pivotal for tumor immune surveillance and eliciting anti-tumor immune responses (Chen et al., 2019). Our analysis, incorporating transcriptomics, single-cell omics, and spatial omics, identified FCN1 as a potential pan-cancer biomarker for macrophage infiltration. Notably, FCN1 exhibited a significant positive correlation with monocyte and macrophage infiltration, indicative of its crucial role in modulating the immune response in tumor patients and shaping the tumor microenvironment. FCN1’s impact on tumor development likely involves its regulatory influence on tumor-associated inflammatory responses. FCN1 expression positively correlates with various immune regulatory factors, including immunosuppressive factors and chemokines, suggesting its ability to modulate immune system function, thereby impacting tumor onset and treatment efficacy. The anticancer immune cycle delineates the pivotal stages of the immune system’s battle against cancer (Chen and Mellman, 2013). In the high-expression group of FCN1, multiple steps of the anti-cancer immune response were significantly upregulated, likely attributable to FCN1’s pro-inflammatory effects within tumors, which activate and regulate the immune system, thereby fostering an anti-cancer immune response. The study by Wang et al. highlighted that FCN1+Tumor-associated macrophages were strongly associated with inflammation induction, which is consistent with our study (Wang et al., 2024). We postulate that increased FCN1 expression augments the inflammatory response within tumors, thereby facilitating antigen release and presentation by cancer cells. Upregulation of FCN1 potentially enhances immune cell activation (e.g., CD4 T cells, CD8 T cells, NK cells, Th1 cells, and MDSC) and their infiltration into tumors by modulating the expression of chemokines, their receptors, and cell adhesion molecules in the tumor microenvironment. Validation of our analysis revealed a higher T cell inflammation score in the FCN1 high-expression group, further affirming our findings. AML patients with elevated FCN1 expression exhibited lower TIDE scores, indicative of enhanced efficacy and prolonged survival with ICB therapy, underscoring FCN1’s role in modulating tumor inflammation and immune responses by regulating immune cell infiltration, expression of immune regulatory factors, and tumor-associated immune activity.

The stemness score is closely associated with tumor aggressiveness and treatment response (Zhang et al., 2023). Patients with elevated stemness scores often display inferior responses to immunotherapy, potentially stemming from diminished immune cell infiltration in the tumor microenvironment. Our findings unveiled a notable negative correlation between FCN1 expression and the stemness score in AML, indicating that tumor cells with reduced FCN1 expression harbor heightened stemness and may develop resistance to treatment. We investigated the interplay between FCN1 and drug sensitivity using GDSC2 data and pinpointed 17 promising compounds, with trametinib and selumetinib emerging as the top contenders. Both compounds act as inhibitors of the ERK-MAPK signaling pathway, implying a potential linkage between FCN1 and this pathway, thereby influencing drug sensitivity. This association offers valuable insights into the intricate functionality of FCN1 and its implications for disease treatment.

We assessed the potential mechanisms underlying FCN1 action in AML. Functional enrichment analysis revealed significant associations between FCN1 and multiple immune and cancer-related pathways. These findings underscore FCN1’s crucial roles in immune regulation, inflammation modulation, and cytokine signaling, pivotal for normal immune function and inflammation regulation. GSEA further demonstrated significant enrichment of FCN1 in various pathways implicated in AML pathogenesis, encompassing apoptosis regulation, cell proliferation, differentiation, and cell survival, alongside its involvement in immune response regulation and inflammation. Studies from liver cancer also highlighted the significant association between FCN1 and its immune response and apoptosis (Sun et al., 2022). To validate these findings, we conducted *in vitro* experiments. Knockdown of FCN1 led to decreased cell proliferation, inhibited cell cycle progression, and increased apoptosis in U937 cells.

In this study, we comprehensively examined the clinical prognosis, immune signature, treatment response, and underlying molecular mechanisms associated with FCN1 across various cancer types, marking the first investigation of its kind. Our analysis indicates that FCN1 serves as an independent prognostic indicator in AML. It appears to modulate inflammation and immune responses within tumors by influencing immune cell infiltration, expression of immune regulatory factors, and tumor-associated immune activity. Furthermore, knockdown of FCN1 alters proliferation, apoptosis, and cell cycle dynamics in AML cell lines. These findings significantly advance our understanding of FCN1’s molecular functions and mechanisms, as well as its clinical prognostic relevance, thus laying groundwork for future investigations into immunotherapeutic strategies for affected patients.

## Data Availability

The original contributions presented in the study are included in the article/Supplementary Material, further inquiries can be directed to the corresponding authors.
