# Supplementary material for: Multi-omics evaluation of the prognostic value and immune signature of FCN1 in pan-cancer and its relationship with proliferation and apoptosis in acute myeloid leukemia
Source: Front Genet. 2024 Jul 29;15:1425075. doi: 10.3389/fgene.2024.1425075 (PMC11320419; doi:10.3389/fgene.2024.1425075)
Supplement: Supplementary file 2 [file Table1.DOCX]

Supplementary Material

Multi-omics evaluation of the prognostic value and immune signature of FCN1 in pan-cancer and its relationship with proliferation and apoptosis in acute myeloid leukemia

# Supplementary Figure


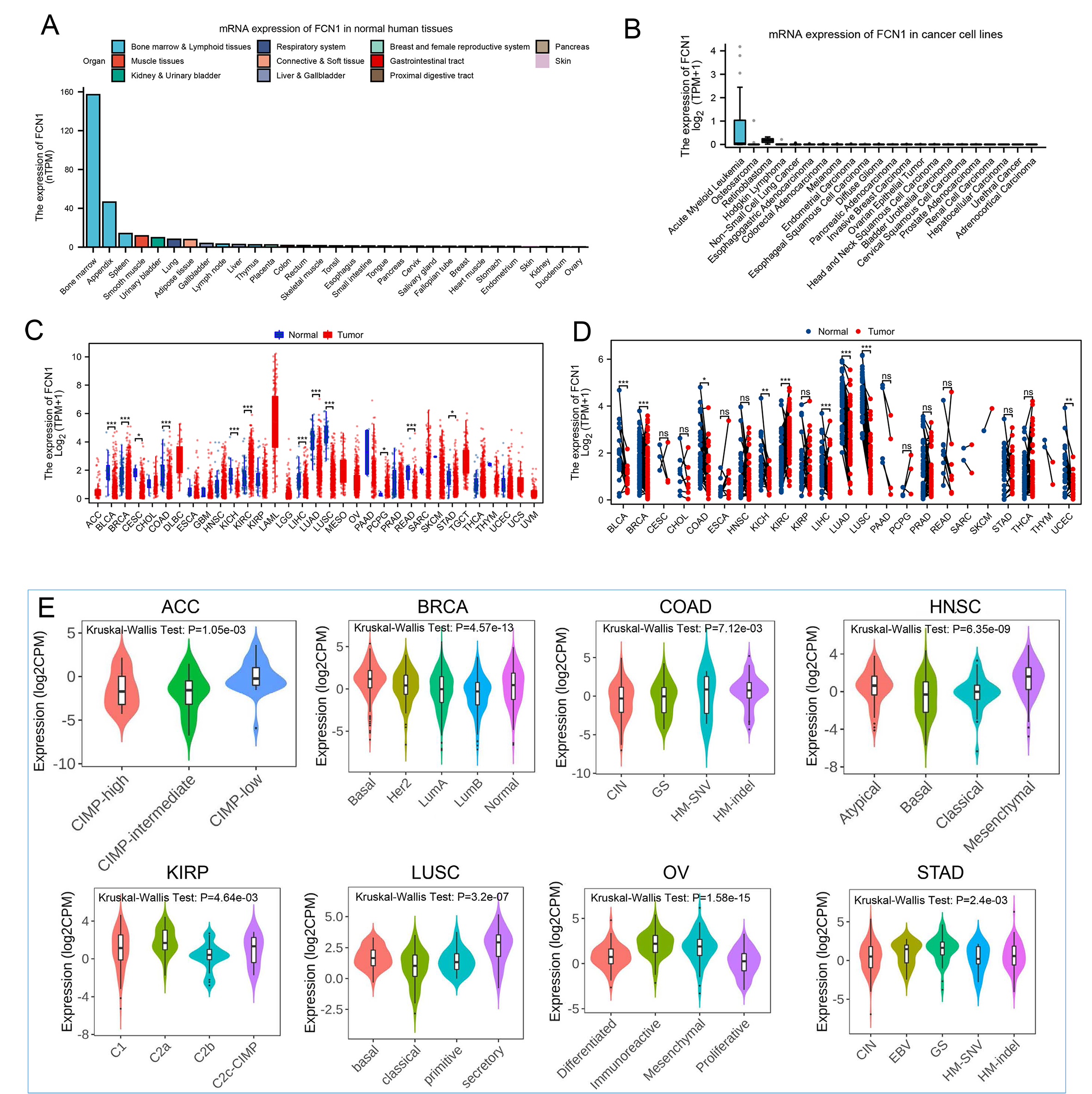


**Supplementary Figure 1.** (A) Expression level of FCN1 in normal tissues (HPA datasets); (B) Expression levels of FCN1 in cancer cell lines (CCLE datasets); (C) Evaluation of differential expression of FCN1 in normal tissues and tumor tissues based on TCGA; (D) TCGA-based assessment of differential expression of FCN1 in paired normal and tumor samples; (E) FCN1 is differentially expressed among multiple cancer subtypes.

**
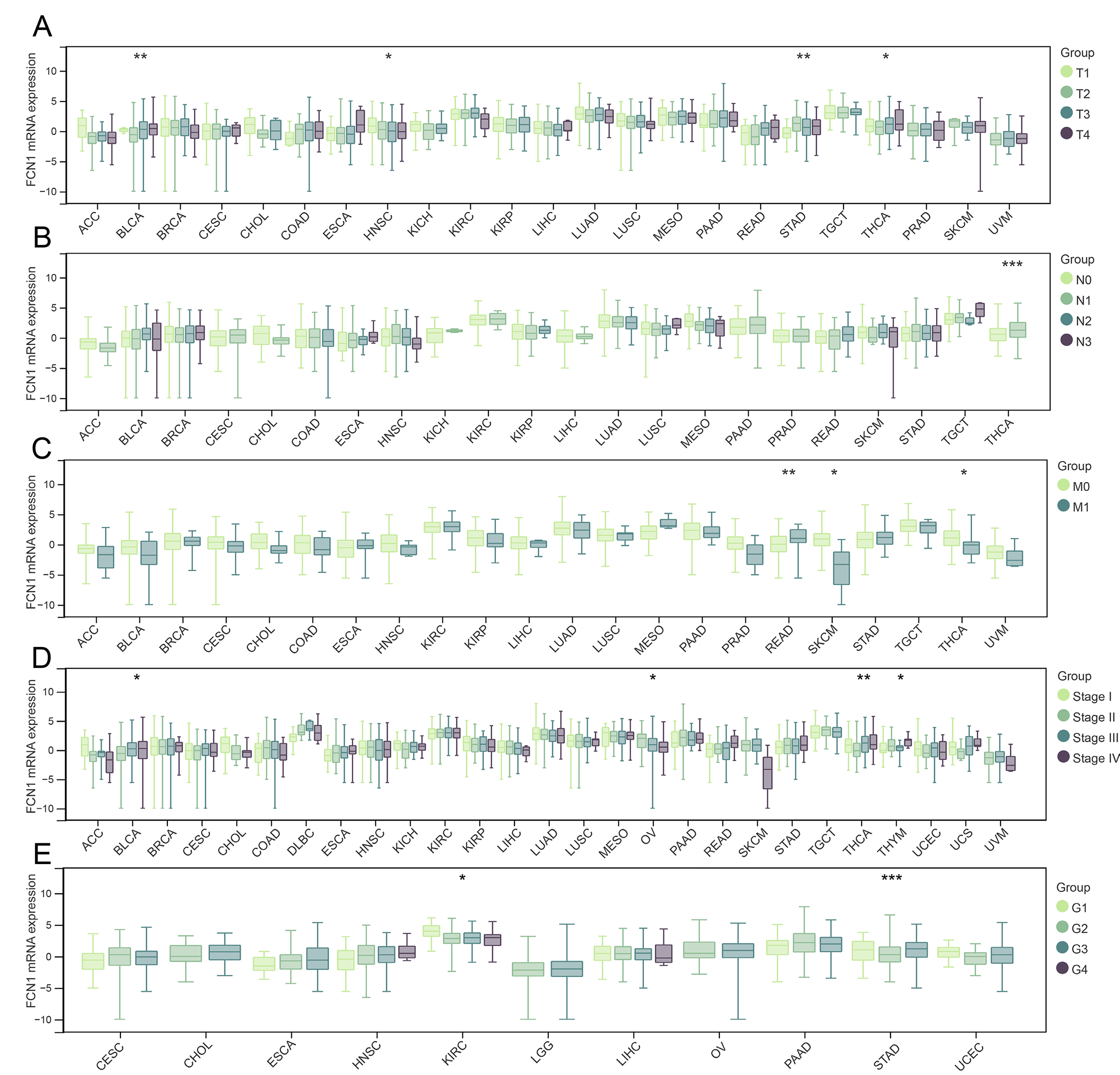
**

**Supplementary Figure 2.** Association between FCN1 expression and pan-cancer clinical T (A), N (B), M (C), Stage (D), Grade (E).

**
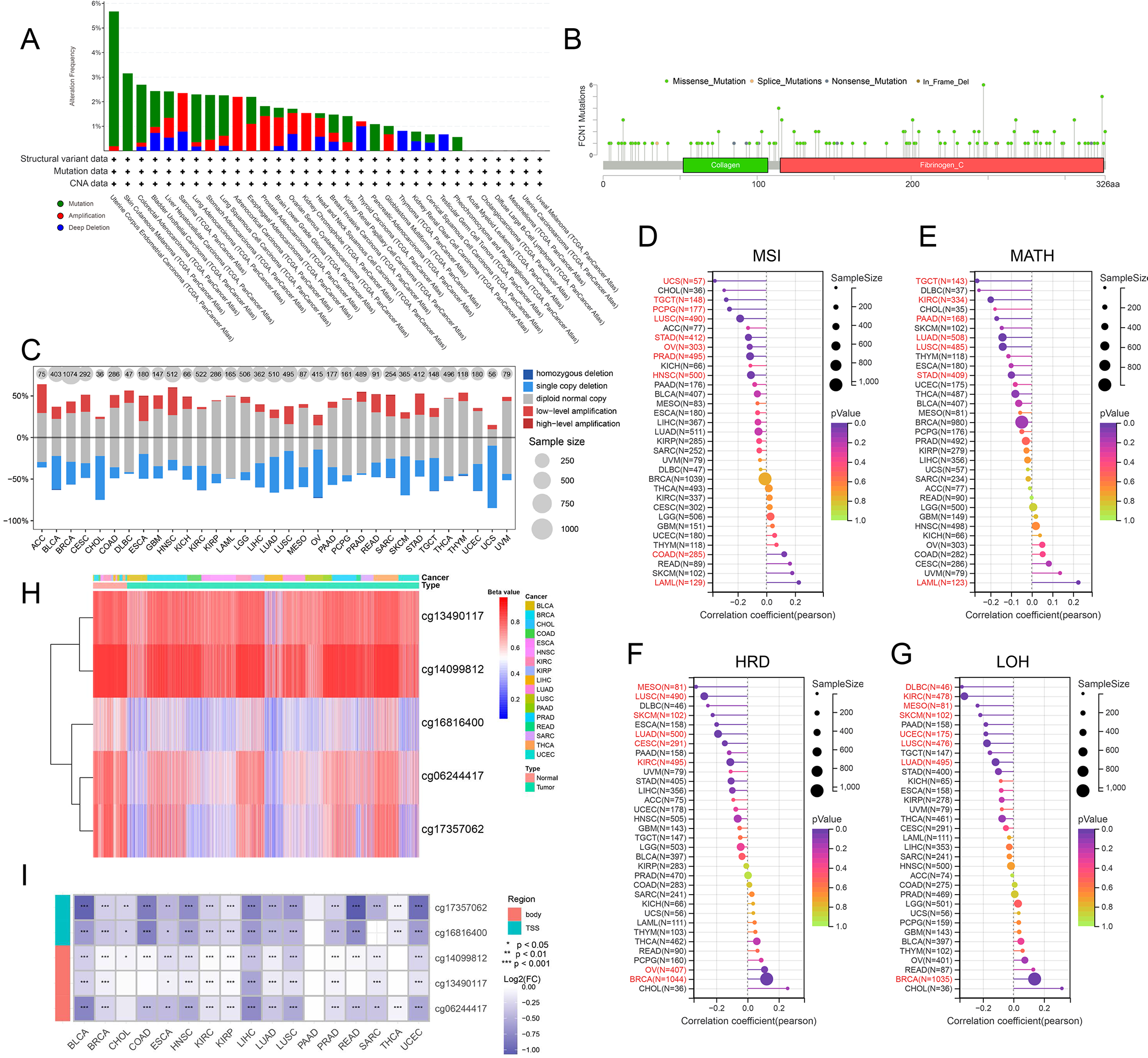
**

**Supplementary Figure 3.** (A) Pan-cancer analyses of genomic changes in FCN1 in the TCGA database were conducted, including analyses of mutations, amplifications, and deep deletions; (B) The pan-cancer FCN1 SNV landscape, including missense, frameshift deletion, and splice site mutations; (C) Copy number variation levels of FCN1 in pan-cancer; (D-G) Lollipop charts were used to visualize correlations between FCN1 levels and MSI (D), MATH (E), HRD (F), and LOH (G), with dot sizes being proportional to sample sizes and dot color being proportional to p-values; (H) Heat map of FCN1 methylation probe levels between pan-cancer normal and tumor samples; (I) Heat map showing differences in FCN1 between normal and tumor tissue;

**
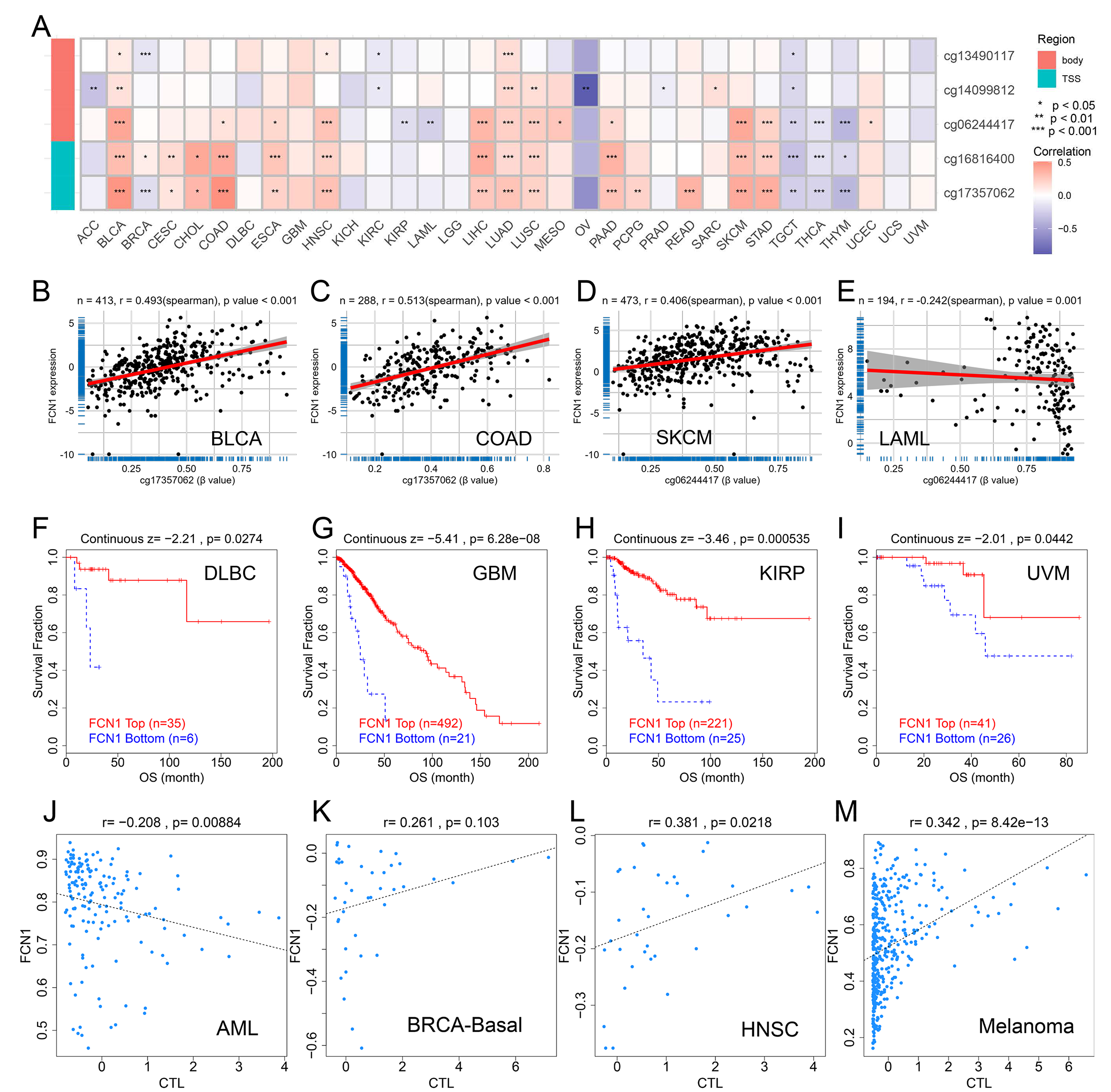
**

**Supplementary Figure 4.** (A) Correlation analysis of FCN1 mRNA expression and methylation in pan-cancer; (B-E) Correlation of FCN1 mRNA expression and methylation in BLCA, COAD, SKCM and LAML; (F-I) The relationship between FCN1 methylation levels and clinical OS; (J-M) Correlation between FCN1 methylation level and CTL.

**
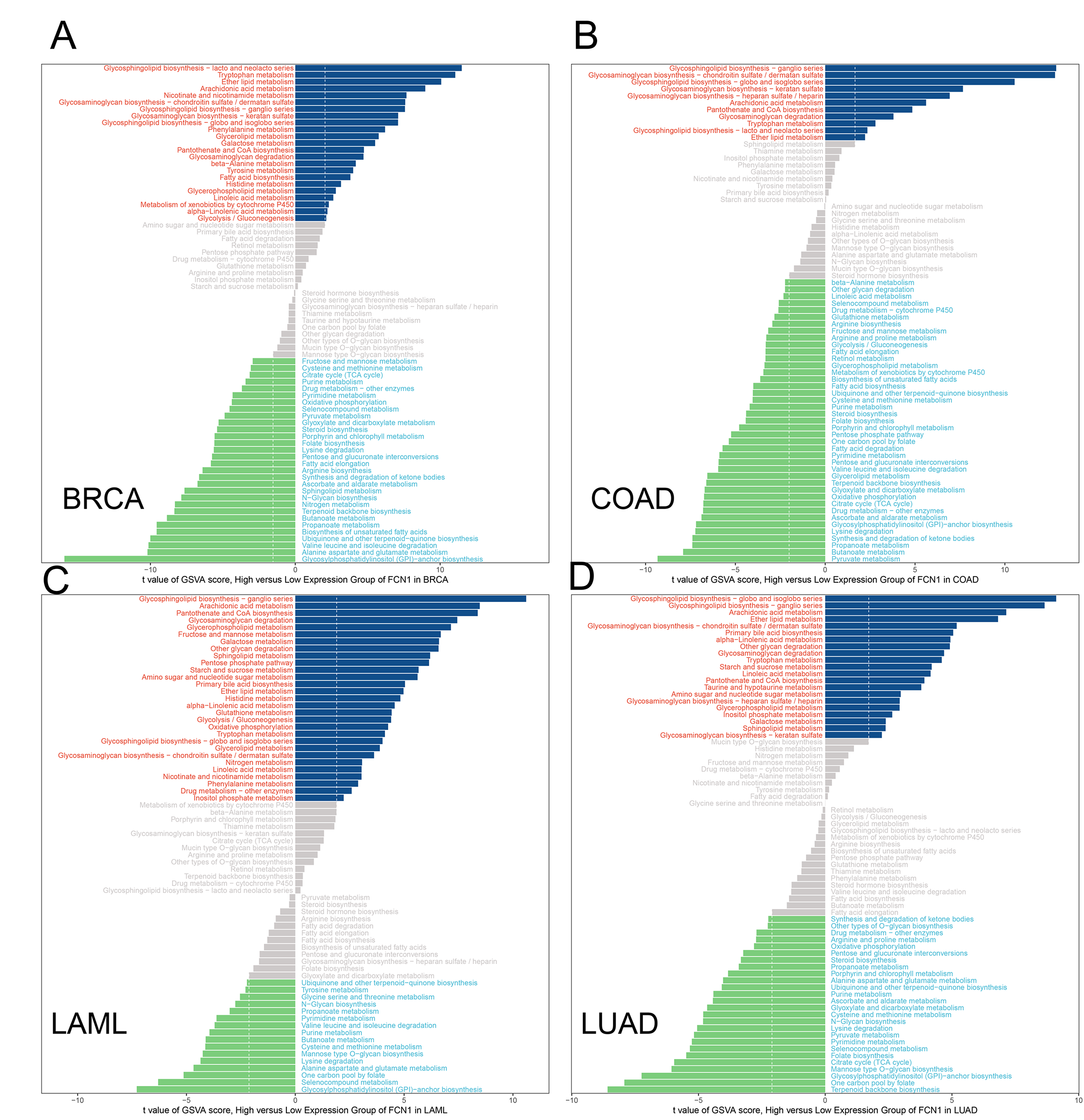
**

**Supplementary Figure 5.** Differences in metabolic pathway GSVA scores between FCN1 high expression group and low expression group in BRCA (A), COAD (B), LAML (C) and LUAD (D).


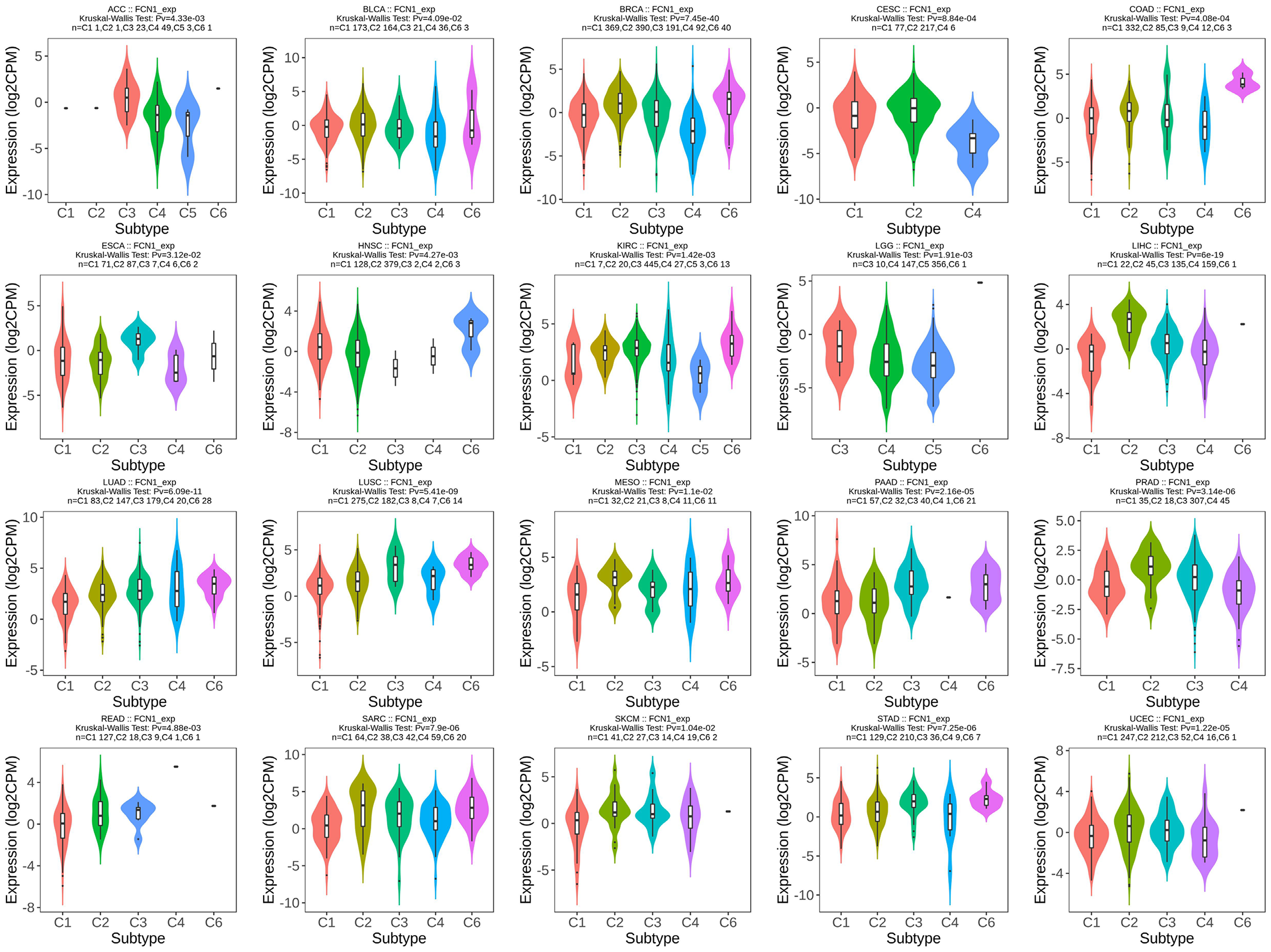


**Supplementary Figure 6.** Differential expression of FCN1 in different immune subtypes in multiple cancers


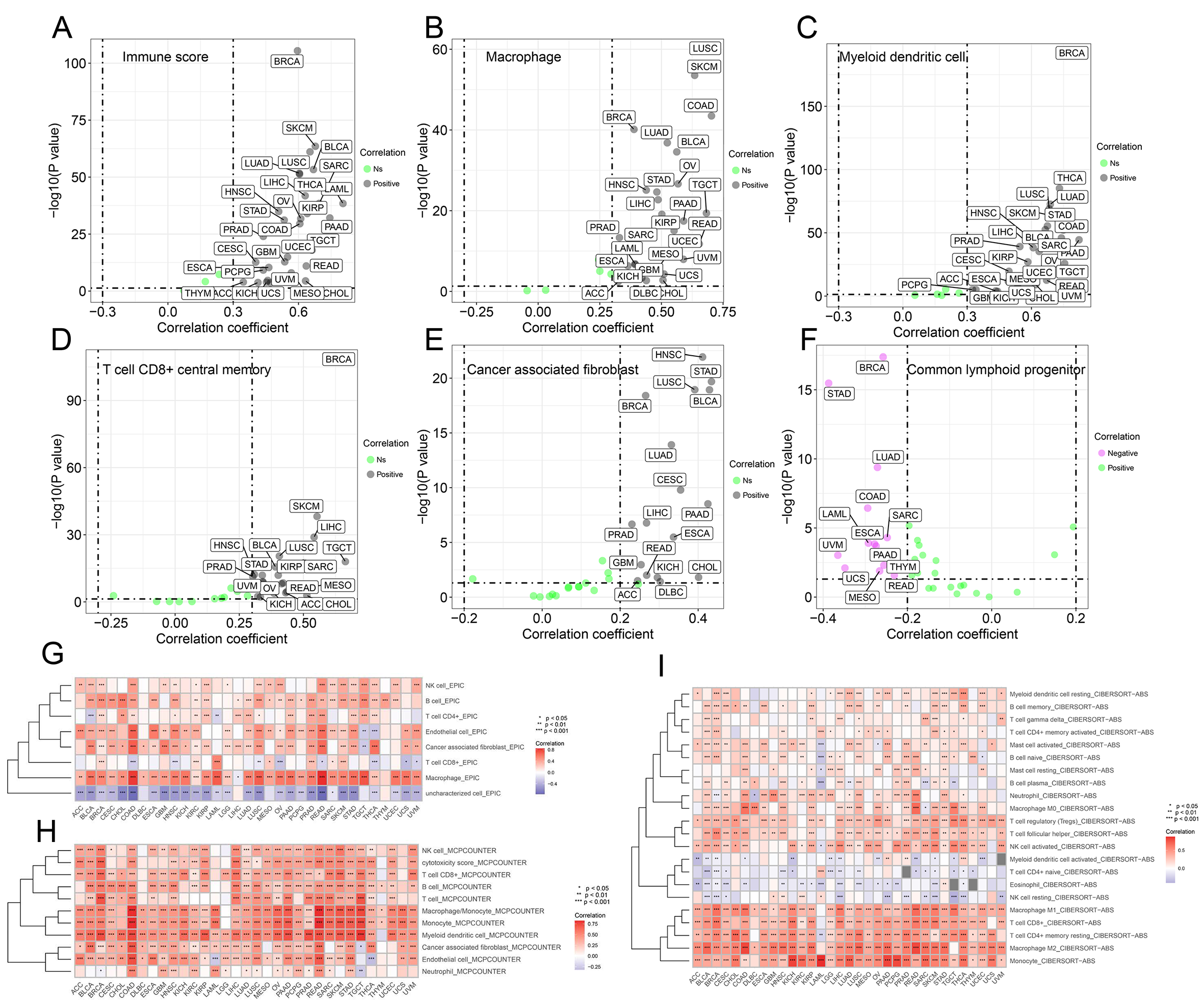


**Supplementary Figure 7.** (A-F) Based on XCELL algorithm to evaluate the correlation between FCN1 and immune score, macrophage infiltration, myeloid dendritic cell infiltration, T cell CD8+ central memory, cancer associated fibroblast and common lymphoid progenitor in pan-cancer; (G-I) Evaluate the correlation between FCN1 and immune cell infiltration in pan-cancer based on EPIC, MCPCOUNTER and CIBERSORT-ABS algorithms.


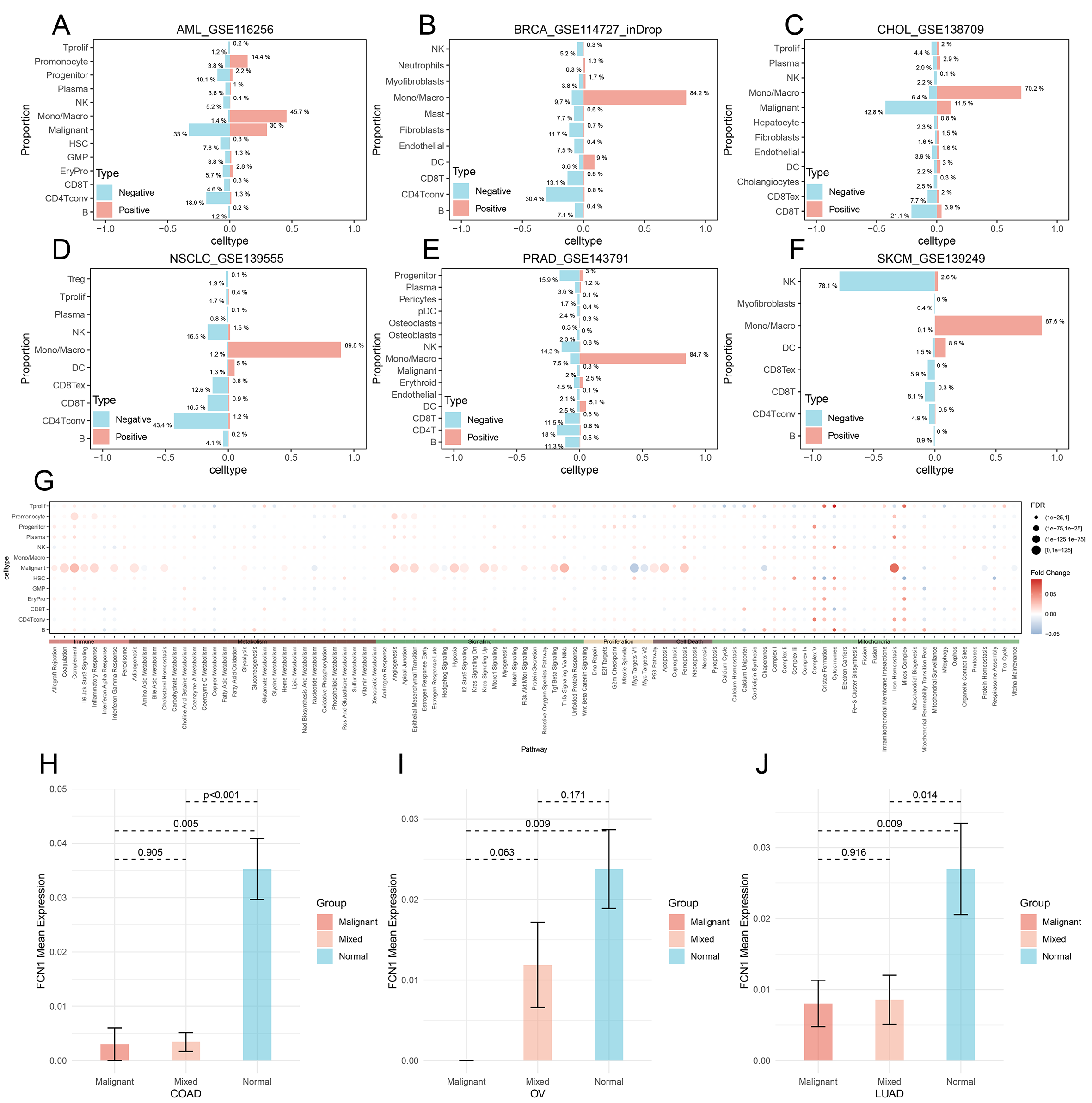


**Supplementary Figure 8.** (A-F) The proportion of each cell type in the FCN1 expression-positive group and the negative group in the single-cell data set; (G) Pathway differences of various cell types in the FCN1 expression positive group and negative group in the AML_GSE116256 data set; (H-J) Differences in the expression of FCN1 in malignant cells, mixed malignant cells and non-malignant cells.

**
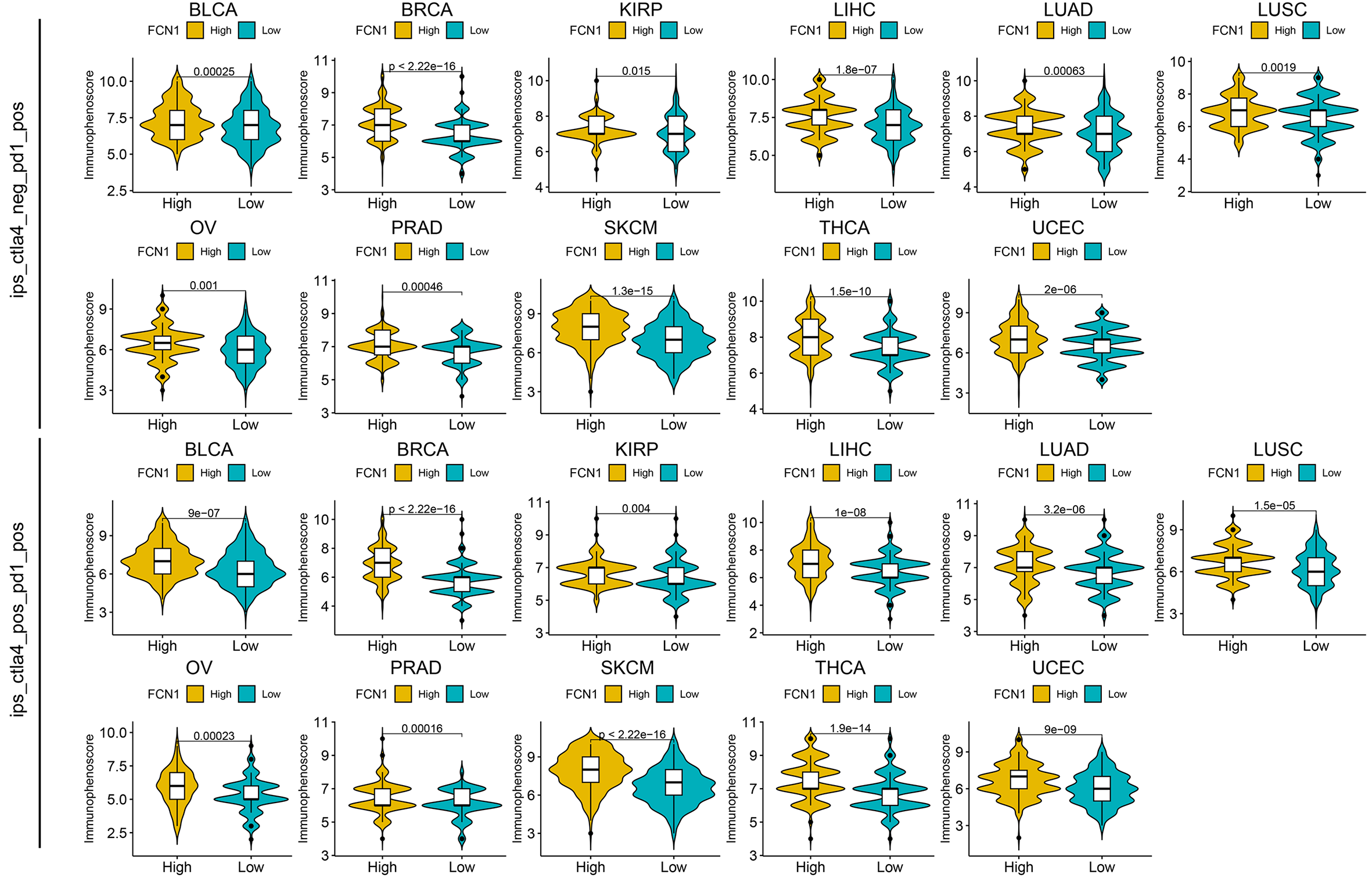
**

**Supplementary Figure 9.** TCIA database evaluates the relationship between FCN1 expression and ips_ctla4_neg_pd1_pos, ips_ctla4_pos_pd1_pos in pan-cancer

**
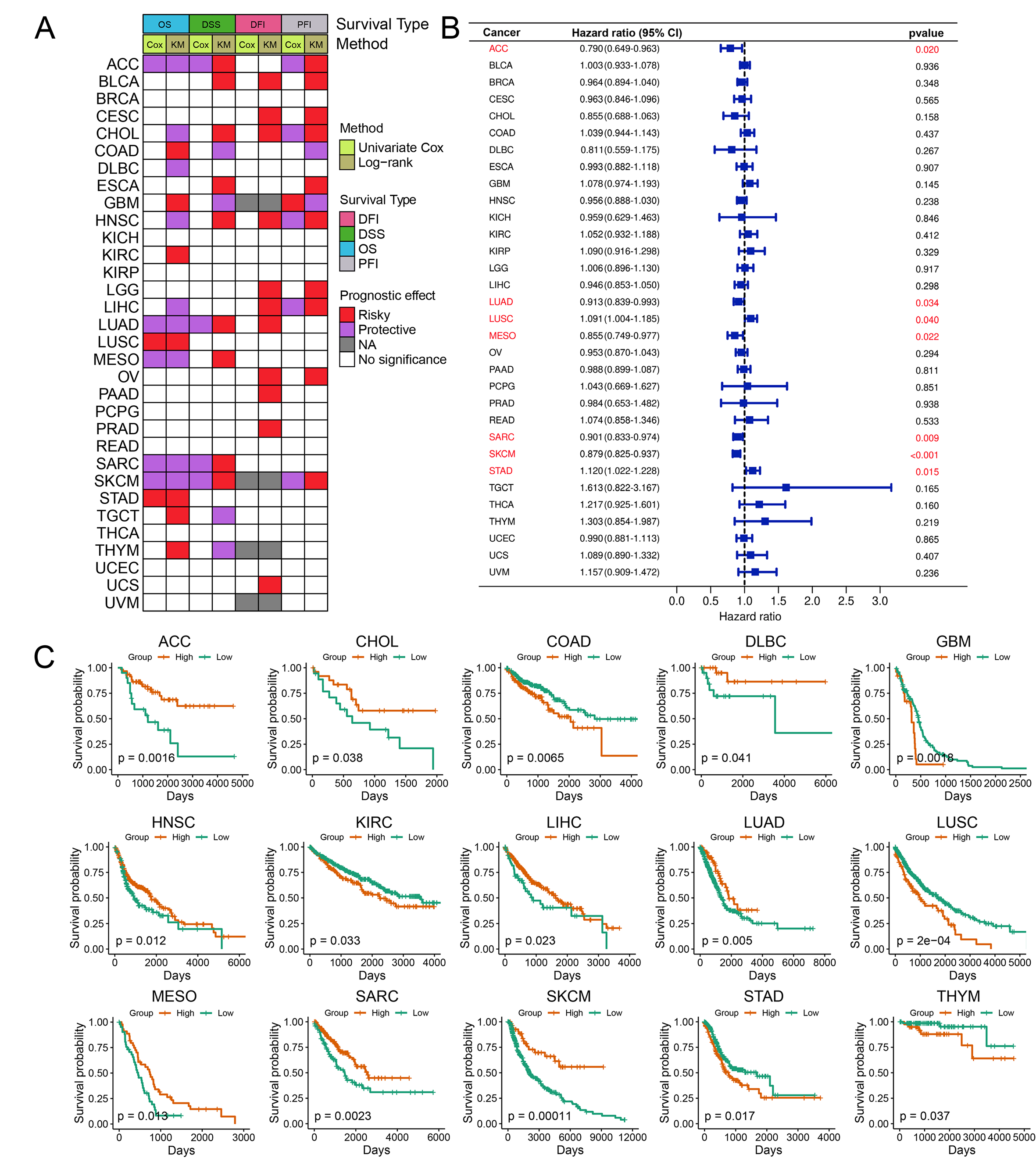
**

**Supplementary Figure 10**. (A) Heatmap showing the correlation between FCN1 expression levels and four survival outcomes, including overall survival (OS), disease-specific survival (DSS), disease-free interval (DFI) and progression-free interval (PFI); (B) Forest plot shows the results of univariate cox analysis of pan-cancer FCN1; (C) KM curves showing significant correlation between FCN1 expression and OS in multiple cancers.**
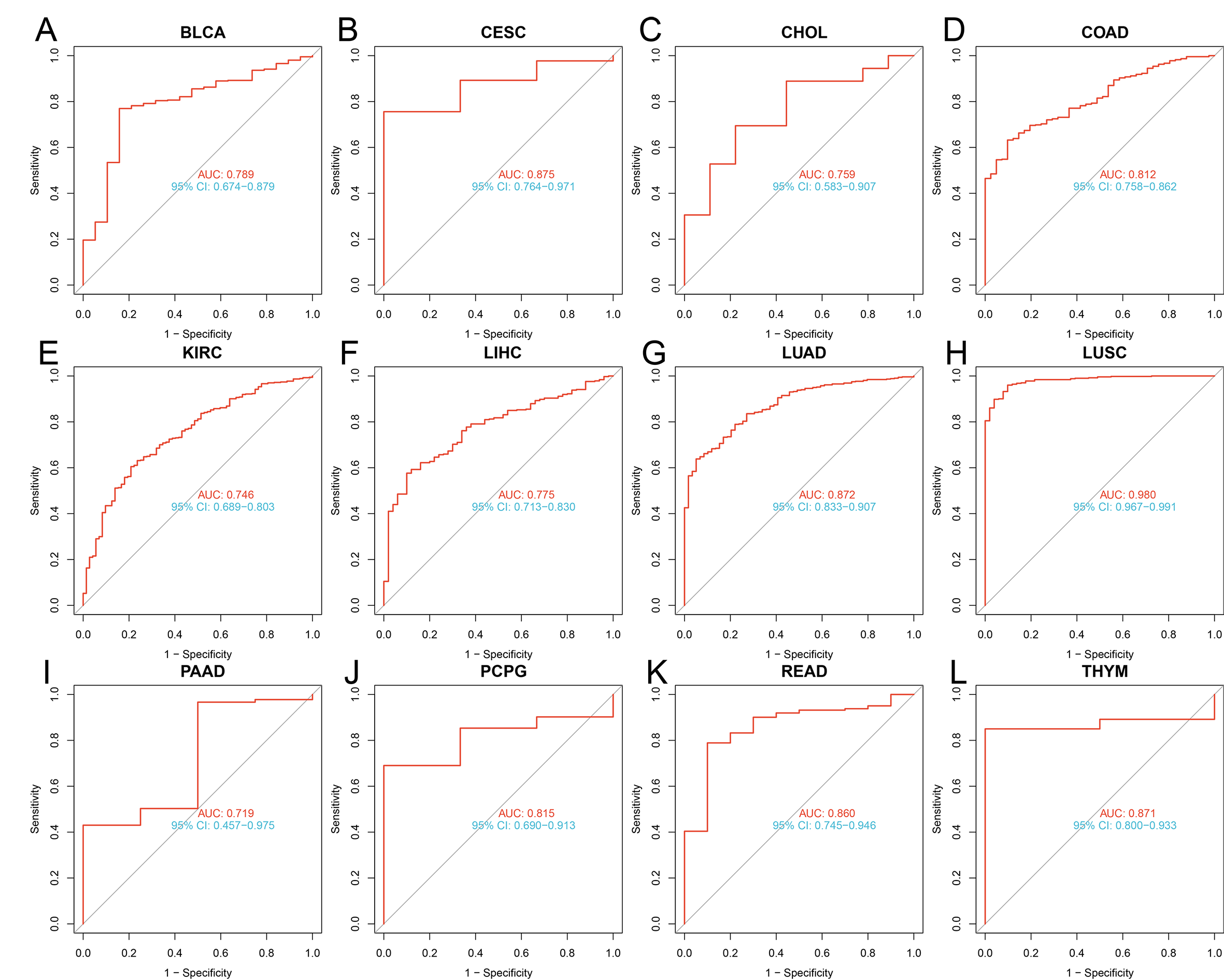
**

**Supplementary Figure 11.** (A-L) ROC analysis evaluates the diagnostic value of FCN1 for cancer.


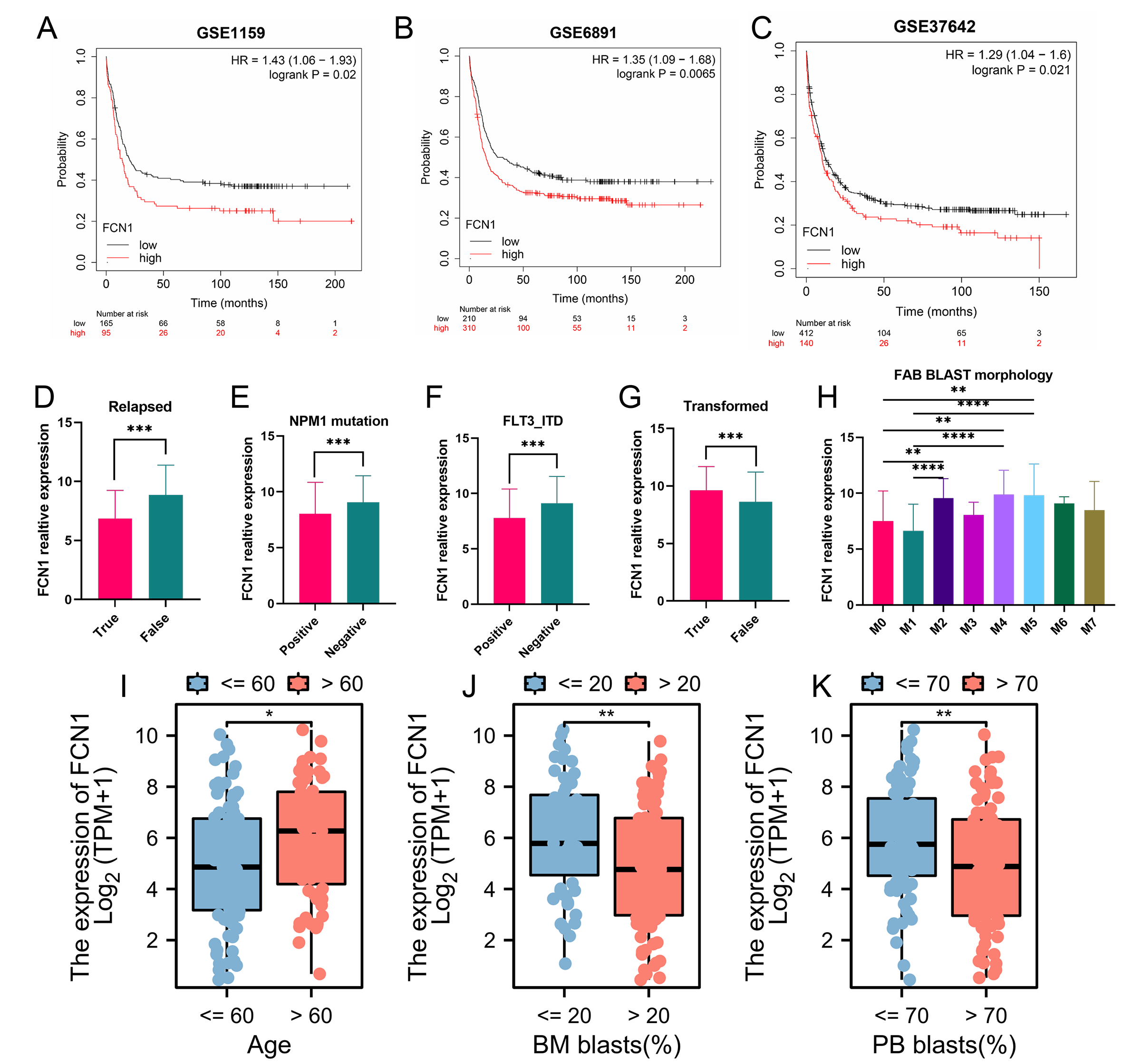


**Supplementary Figure 12.** (A-C) Validation of the prognostic value of FCN1 in AML based on multiple GEO data sets; (D-H) Correlation between FCN1 and relapsed, NPM1 mutation, FLT3_ITD, transformed, FAB blast; (I-K) Correlation between FCN1 and Age, BM blasts, and PB blasts.


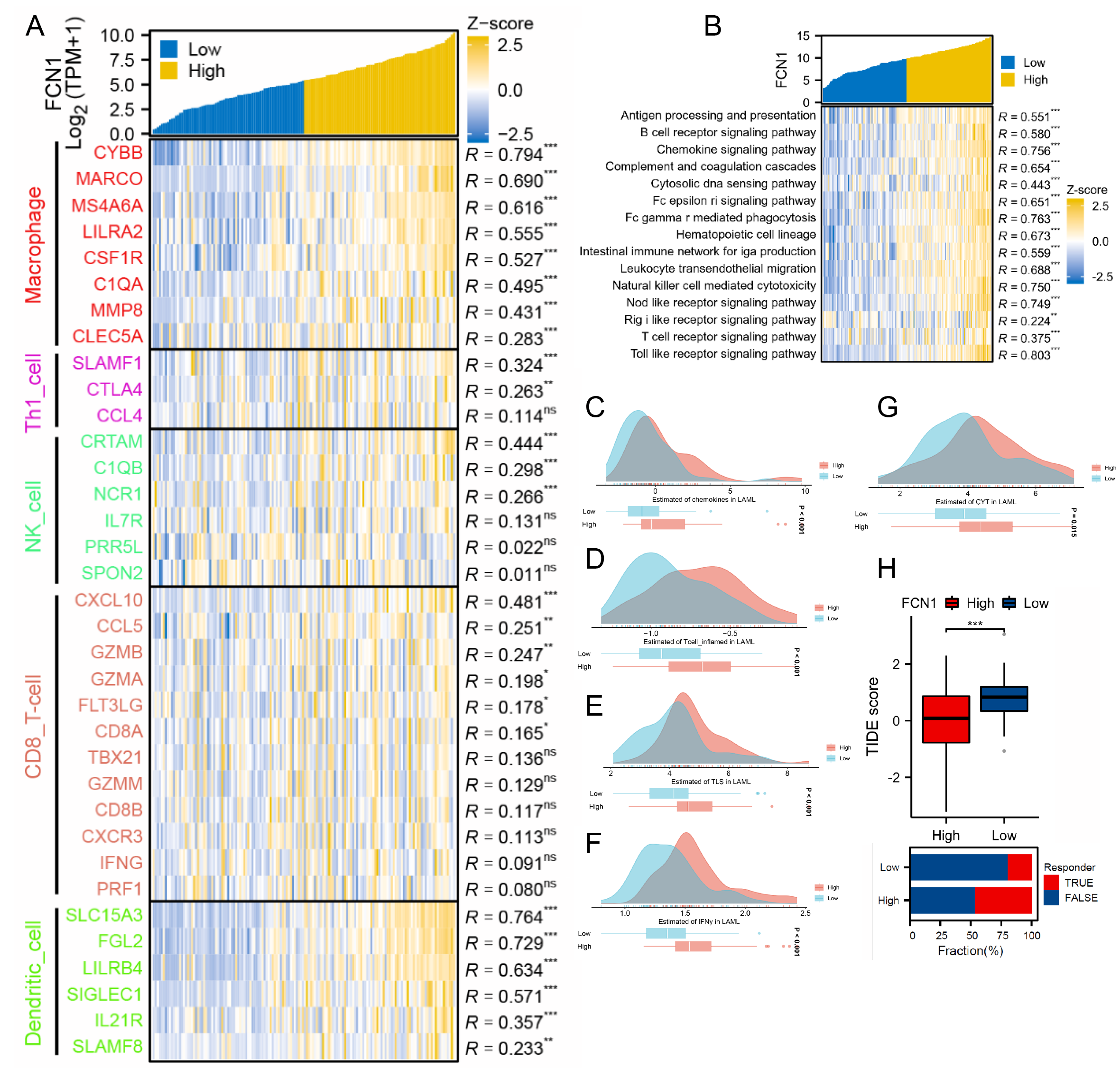


**Supplementary Figure 13**. (A) Correlation between *FCN1* and effector genes of tumor-associated immune cells; (B) FCN1 is significantly positively correlated with multiple inflammation-related pathways in AML; (C-G) Differences in Chemokines score, Tcell_inflamed score, TLS, IFNy and CYT scores in FCN1 high and low expression groups; (H) TIDE scores of FCN1 high and low expression groups.


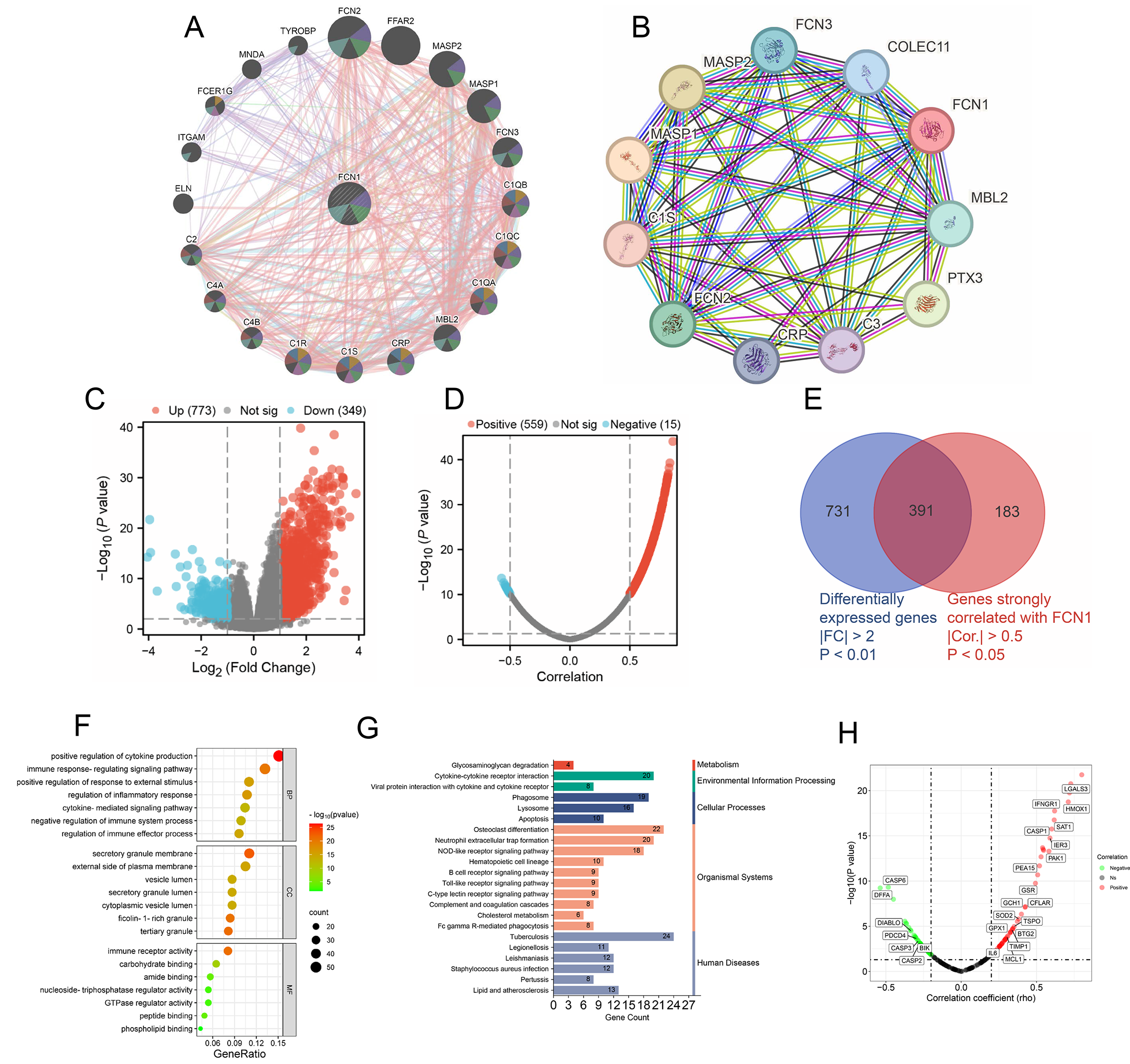


**Supplementary Figure 14.** (A) Evaluation of FCN1-interacting genes based on GeneMANIA; (B) Protein interaction network diagram of FCN1; (C) Identification of differentially expressed genes in AML based on FCN1 high and low expression groups; (D) Genes significantly associated with FCN1 in AML; (E) Venn diagram showing significantly related hub genes of FCN1; (F) GO functional enrichment analysis; (G) KEGG functional enrichment analysis; (H) Correlation analysis of FCN1 and apoptosis-related genes in AML.
